# Oxygen‐Enriched Metal‐Phenolic X‐Ray Nanoprocessor for Cancer Radio‐Radiodynamic Therapy in Combination with Checkpoint Blockade Immunotherapy

**DOI:** 10.1002/advs.202003338

**Published:** 2020-12-31

**Authors:** Wei Sang, Lisi Xie, Guohao Wang, Jie Li, Zhan Zhang, Bei Li, Sen Guo, Chu‐Xia Deng, Yunlu Dai

**Affiliations:** ^1^ Cancer Center Faculty of Health Sciences University of Macau Macau SAR, 999078 China; ^2^ Institute of Translational Medicine Faculty of Health Sciences University of Macau Macau SAR, 999078 China

**Keywords:** hemoglobin, immunotherapy, metal‐phenolic coordination, nanomedicine, radiodynamic therapy, radiotherapy

## Abstract

Radiotherapy (RT) based on DNA damage and reactive oxygen species (ROS) generation has been clinically validated in various types of cancer. However, high dose‐dependent induced toxicity to tissues, non‐selectivity, and radioresistance greatly limit the application of RT. Herein, an oxygen‐enriched X‐ray nanoprocessor Hb@Hf‐Ce6 nanoparticle is developed for improving the therapeutic effect of RT‐radiodynamic therapy (RDT), enhancing modulation of hypoxia tumor microenvironment (TME) and promoting antitumor immune response in combination with programmed cell death protein 1 (PD‐1) immune checkpoint blockade. All functional molecules are integrated into the nanoparticle based on metal‐phenolic coordination, wherein one high‐Z radiosensitizer (hafnium, Hf) coordinated with chlorin e6 (Ce6) modified polyphenols and a promising oxygen carrier (hemoglobin, Hb) is encapsulated for modulation of oxygen balance in the hypoxia TME. Specifically, under single X‐ray irradiation, radioluminescence excited by Hf can activate photosensitizer Ce6 for ROS generation by RDT. Therefore, this combinatory strategy induces comprehensive antitumor immune response for cancer eradication and metastasis inhibition. This work presents a multifunctional metal‐phenolic nanoplatform for efficient X‐ray mediated RT‐RDT in combination with immunotherapy and may provide a new therapeutic option for cancer treatment.

## Graphical abstract

Oxygen‐enriched X‐ray nanoprocessor Hb@Hf‐Ce6 nanoparticles are developed for improving therapeutic effect of radiotherapy‐radiodynamic therapy, enhancing modulation of hypoxia tumor microenvironment, and promoting antitumor immune response in combination with anti‐PD‐1 antibody. This work presents the favorable metal‐phenolic X‐ray nanoprocessor, which could open new avenues for cancer treatment based on nanomedicine.

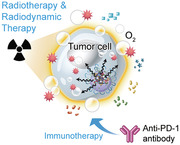

## 1 Introduction

Although significant advances have been made in fighting tumors in recent years, the failure of conventional therapies to eradicate tumors completely leads to tumor metastasis.^[^ ^1^ ^]^ Radiotherapy (RT) with many irreplaceable advantages occupies a promising approach in cancer therapy.^[^ ^2^ ^]^ However, RT suffers from hypoxia‐associated radioresistance greatly induced by complicated tumor microenvironment (TME).^[^ ^3^ ^]^ To combat this obstacle, substantial efforts have been made to improve the oxygen levels in TME. One promising approach is to delivery oxygen directly.^[^ ^4^ ^]^ Hemoglobin (Hb), as oxygen‐transport metalloprotein in the red blood cells, can be employed as a candidate for oxygen delivery owing to its safe and effective oxygen‐carriers capability. However, free Hb molecules fail to be employed directly as oxygen provider due to their nephrotoxicity and immunogenicity.^[^ ^5^ ^]^ To address this issue, a promising strategy is to encapsulate or hybridize the Hb into the nanoparticle frameworks, which greatly reduces not only the toxicity but also immunogenicity without negative impact to Hb's oxygen‐carrying function.^[^ ^6^ ^]^ Moreover, the inherent immunomodulatory adjuvant feature of RT boosts the immunotherapeutic performance of programmed cell death protein 1 (PD‐1) checkpoint blockade.^[^ ^7^ ^]^

Given the special characteristics of self‐assembly coordination between metal ions and phenolic compounds, metal‐phenolic networks (MPNs) exhibit specific functional moieties, redox‐responsive behavior, and reversible assemble properties.^[^ ^8^ ^]^ Therefore, as an emerging new class of coordination materials, the MPNs‐based nanomaterials have been explored as multifunctional nanoplatforms for various biomedical applications.^[^ ^9^ ^]^ Gallic acid (GA), one of the natural polyphenols, can chelate various metal ions.^[^ ^10^ ^]^ Chelating high‐Z metal ions with phenolic agents would give a good opportunity for nano‐radiosensitizers formation to improve RT.^[^ ^11^ ^]^ Since high‐Z metals could promote radiation dose enhancement,^[^ ^12^ ^]^ Hafnium (Hf), a promising high‐Z metal, exhibits superior photoelectric effect upon X‐ray excitation that can not only be used as a radiosensitizer, but also be employed as a scintillator for radioluminescence, which can be utilized as the favorable light source for radiodynamic therapy (RDT) to break the depth limitation of laser source by traditional photodynamic therapy (PDT).^[^ ^13^ ^]^ Therefore, abundant reactive oxygen species (ROS) can be generated for tumor eradication.^[^ ^14^ ^]^ RDT, as the “photonic missile,” can not only improve the RT effect to the specific tumor area, but also increase the production of ROS in the TME, so as to eliminate tumor cells accurately by an omnidirectional and multiangular manner.^[^ ^15^ ^]^ RDT has achieved remarkable effects in the treatment of advanced cancer patients with recurrence, spread, and multiple metastasis.^[^ ^16^ ^]^ The combination of RT‐RDT and checkpoint blockade immunotherapy based on nanoplatform may realize intriguing synergistic effects for cancer treatment and prevention.^[^ ^14^ ^]^

**Figure 1:**
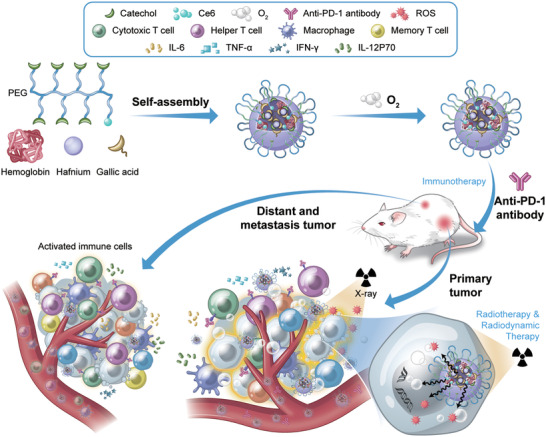
Schematic illustration showed the Hb@Hf‐Ce6 nanoparticles‐mediated X‐ray induced radiotherapy‐radiodynamic therapy‐immunotherapy for the eradication of both primary and distant tumors. Hb was encapsulated in the Hf‐phenolic coordination nanoplatform for oxygen delivery through self‐assembly. A mouse orthotopic and bilateral tumor model was prepared to evaluate the efficacy of combination therapy based on Hb@Hf‐Ce6 nanoparticles for the both primary and distant tumors. Hf is employed as the radiosensitizer to promote radiation dose enhancement and improve ROS production to inhibit the growth of the primary tumors. The PD‐1 checkpoint blockade solved the issues of low immune response rate caused by radiotherapy‐radiodynamic therapy, which led to a distant and long‐term therapeutic efficacy. Meanwhile, the activated immune cells and immune cytokines combated the distant and metastasis tumors.

Herein, we developed a facile X‐ray nanoprocessor (Hb@Hf‐Ce6 NPs) based on metal‐phenolic coordination for oxygen delivery and X‐ray‐triggered ultrasensitive ROS generation in tumor region to overcome immunosuppressive TME and achieve enhanced tumor inhibition by combination of RT‐RDT integrated with checkpoint blockade immunotherapy (**Figure** **1**). In this specific nanoplatform, chlorin e6 (Ce6)‐polyethylene glycol (PEG)‐polyphenols were synthesized by conjugating a photosensitizer Ce6 and catechol on 8‐arm PEG. Then, the spherical Hb@Hf‐Ce6 NPs were fabricated via self‐assembly coordination of Hf and Ce6‐PEG‐polyphenols with Hb encapsulation for oxygen delivery, in which the Ce6‐PEG‐polyphenols coordination network protects Hb from exposure to biological environment during blood circulation and prolongs its stability and circulation half‐life. The obtained Hb@Hf‐Ce6 NPs not only enhanced the RT effect but also activated Ce6 with the assistance of efficient radioluminescence for RDT. Oxygen released from Hb@Hf‐Ce6 NPs in the tumor site can be exploited as ROS donors and modulate hypoxia‐induced radioresistance. In situ dying tumor cells induced by RT‐RDT significantly activated the antitumor immunity in primary tumor and a great abscopal effect to distant tumor was achieved without light exposure and metastatic tumor by combining PD‐1 checkpoint blockade immunotherapy. Our work demonstrates a smart metal‐phenolic X‐ray nanoprocessor with super‐additive synergistic for cancer therapy.

## 2 Results

### 2.1 The Preparation and Characterization of Hb@Hf‐Ce6 NPs

**Figure 2:**
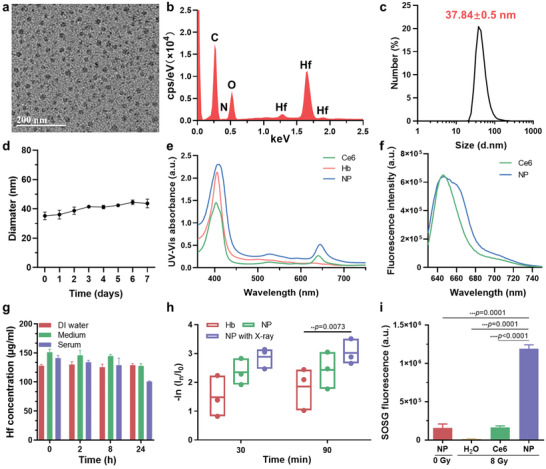
Characterizations of Hb@Hf‐Ce6 NPs. a) Transmission electron microscopy (TEM) images of Hb@Hf‐Ce6 NPs. b) Energy‐dispersive X‐ray spectroscopy (EDS) analysis data of Hb@Hf‐Ce6 NPs. c) The size of Hb@Hf‐Ce6 NPs determined by dynamic light scattering (DLS). d) Diameters of Hb@Hf‐Ce6 NPs stored in DI water in 7 days. e) The UV–vis absorption spectra of chlorin e6 (Ce6), hemoglobin (Hb), and Hb@Hf‐Ce6 NPs (NP). f) Fluorescence intensity of chlorin e6 (Ce6) and Hb@Hf‐Ce6 NPs (NP). g) The stability of Hb@Hf‐Ce6 NPs in DI water, 1640 medium and serum at various timepoints (0, 2, 8, and 24 h). h) The oxygen release behavior of hemoglobin (Hb) and Hb@Hf‐Ce6 NPs (NP) with/without X‐ray irradiation was evaluated by Ru(bpy)_3_Cl_2_ probe. Hb and NP were oxygenated previously. i) Fluorescence intensity of ^1^O_2_ generation was measured by singlet oxygen sensor green (SOSG). Data are presented as mean values ± SD (*n* = 3).

The phenolic compounds are good candidates for coordination with specific metal ions. Polyphenol derivatives often inherit excellent properties from functional molecules. Herein, a polyphenol derivative, Ce6‐PEG‐polyphenols was first synthesized following a reported protocol with some modification.^[^ ^17^ ^] 1^H NMR spectrums were performed to confirm the composition of Ce6‐PEG‐polyphenols, suggesting the polyphenol derivative was successfully prepared (Figure S1, Supporting Information). The molar ratio of Ce6, PEG, and catechol was 1:1:7 in the Ce6‐PEG‐polyphenols, providing enough Ce6 for RDT. In addition, GA was applied as natural polyphenol to chelate Hf ions for further RT enhancement. A facile strategy of Hb@Hf‐Ce6 NPs fabrication is illustrated in Figure 1. Hf, Ce6‐PEG‐polyphenols, GA, and Hb can form nanoparticles rapidly via self‐assembled coordination between Hf and polyphenols. The monodispersed Hb@Hf‐Ce6 NPs with size about 30 nm can be clearly observed in transmission electron microscopy (TEM) images with different magnifications (**Figure** **2**a; Figure S2, Supporting Information). Energy dispersive X‐ray spectroscopy (EDX) analysis of Hb@Hf‐Ce6 NPs demonstrated that C, N, O, and Hf were distributed in the Hb@Hf‐Ce6 NPs, indicating the Hf has been integrated in Hb@Hf‐Ce6 NPs successfully (Figure 2b). The Hf distribution in an individual nanoparticle was further confirmed by high‐angle annular dark‐field scanning transmission electron microscopy (HAADF‐STEM) with elemental mapping, indicating the Hf has been integrated in Hb@Hf‐Ce6 NPs successfully (Figure S3, Supporting Information).The Hf ratio in Hb@Hf‐Ce6 NPs was as high as 29.04 wt% by inductively coupled plasma mass spectrometry (ICP‐MS). High concentration of Hf is conducive to the effective RT‐RDT. The size of Hb@Hf‐Ce6 NPs was further investigated by dynamic light scattering (DLS) with dynamic size 37.84 ± 0.5 nm (Figure 2c), which was consistent with the TEM images. Furthermore, the stability of Hb@Hf‐Ce6 NPs in DI water was monitored (Figure 1d). The size of Hb@Hf‐Ce6 NPs did not show significant change, which proved that Hb@Hf‐Ce6 NPs were stable in DI water for at least 7 days. The UV–vis absorption spectra showed that characteristic peaks of both Hb and Ce6 can be found in Hb@Hf‐Ce6 NPs, indicating the successful preparation of Hb@Hf‐Ce6 NPs (Figure 2e). The successful synthesis of Hb@Hf‐Ce6 NPs was further verified by fluorescence spectroscopy (Figure 2f), the emission wavelengths of free Ce6 and Ce6 in the Hb@Hf‐Ce6 NPs were similar under the 620 nm excitation.

The stability in different physiological buffers, O_2_ dissociation and release ability, and ^1^O_2_ generation capability of Hb@Hf‐Ce6 NPs were evaluated in vitro. First, ICP‐MS was applied to measure the Hf concentration when Hb@Hf‐Ce6 NPs exposed to various kinds of solutions, including DI water, 1640 complete medium and serum from 0 to 24 h. As given in Figure 2g, there was no obvious difference of Hf concentration in various timepoints, indicating that Hb@Hf‐Ce6 NPs were stable in various physiological environments. Second, the dissolved O_2_ by free Hb and Hb@Hf‐Ce6 NPs was measured by an oxygen detector at an interval of 300 s (Figure S4, Supporting Information). The O_2_ release curve and rate in Hb@Hf‐Ce6 NPs were consistent with the free Hb group. At 300 s, the dissolved O_2_ of Hb@Hf‐Ce6 NPs and free Hb reached 30.2 and 27.7 mg L^−1^, respectively. It confirmed that there was no significant influence in oxygen‐carrying capacity after the encapsulation of Hb in Hb@Hf‐Ce6 NPs. Furthermore, O_2_ release behavior of Hb@Hf‐Ce6 NPs was measured by a Ru(bpy)_3_Cl_2_ probe, which can be exploited as a dissolved oxygen sensor. The test was performed at two timepoints (30 and 90 min, Figure 2h). The amount of oxygen loaded in Hb@Hf‐Ce6 NPs was higher than free Hb, which indicated that Hb@Hf‐Ce6 NPs could enhance the oxygen loading ability of Hb and control O_2_ release in the target sites. Significantly, under external X‐ray irradiation, more O_2_ can release, suggesting that X‐ray can trigger O_2_ release from Hb@Hf‐Ce6 NPs. In order to verify the singlet oxygen (^1^O_2_) generation efficiency of Ce6, singlet oxygen sensor green (SOSG) was used to monitor the ^1^O_2_ generation (Figure 2i). Interestingly, compared to Hb@Hf‐Ce6 NPs without X‐ray irradiation, high level of ^1^O_2_ can be produced from Hb@Hf‐Ce6 NPs under 8 Gy X‐ray irradiation. Notably, the fluorescence intensity of SOSG from Hb@Hf‐Ce6 NPs group under X‐ray irradiation was 7.29 times higher than that of free Ce6. Therefore, it clearly demonstrated that Hf ions in Hb@Hf‐Ce6 NPs possessed an excellent capability to transfer X‐ray to optical luminescence and initiate generation of ^1^O_2_ by Ce6. Owing to its excellent O_2_‐carrying capacity, X‐ray responsive O_2_ release, and high X‐ray triggered ^1^O_2_ generation efficiency, the nanoplatform can be used as a promising candidate for further in vitro and in vivo antitumor experiment.

### 2.2 In Vitro Therapeutic Efficacy of Hb@Hf‐Ce6 NPs

**Figure 3:**
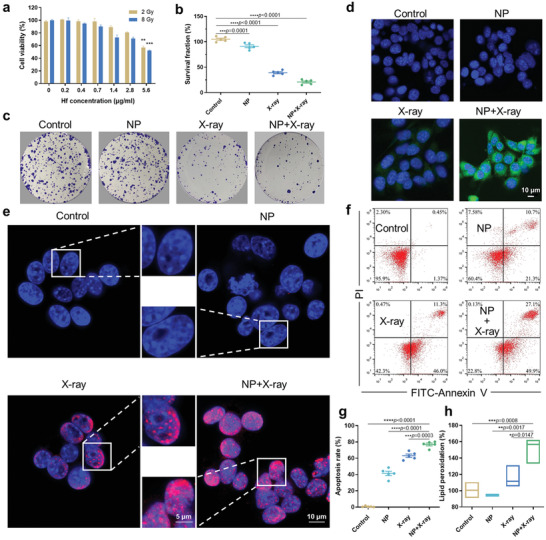
In vitro therapeutic efficacy of Hb@Hf‐Ce6 NPs. a) Cytotoxicity of Hb@Hf‐Ce6 NPs on 4T1 cells with X‐ray irradiation by MTT assay. b,c) Statistics and respective images of colony formation of 4T1 cells after treated with Hb@Hf‐Ce6 NPs (NP) and/or X‐ray irradiation (8 Gy). Surviving fraction was calculated as (surviving colonies) / (cells seeded × plating efficiency). d) Reactive oxygen species (ROS) generation was measured through the 2′,7′‐dichlorofluorescin diacetate (DCFH‐DA) method by confocal laser scanning microscope (Nikon A1R). e) DNA double‐strand breaks in 4T1 cells treated with PBS or NP with/without X‐ray irradiation (8 Gy). The nuclei were stained by Hoechst 33342 (blue) and *γ*‐H2AX (red). f,g) Annexin V/PI analysis of 4T1 cells incubated with 1640 medium or NP with/without X‐ray irradiation (8 Gy) and quantification of late apoptotic cells plus necrotic cells. h) Lipid peroxidation levels of RT‐RDT evaluated by a Malondialdehyde (MDA) assay kit. Data are presented as mean values ± SD (*n* = 5). **p < 0.01, ***p < 0.001.

MTT assay of Hb@Hf‐Ce6 NPs was carried out on three types of tumor cell lines, including B16F10 melanoma cell, MC38 colon carcinoma cell, and 4T1 mammary carcinoma cell (Figure S5, Supporting Information). Cell viabilities of all three cell lines were higher than 80% even at a high Hf concentration (11.2 µg mL^−1^) after 48 h incubation, indicating that Hb@Hf‐Ce6 NPs showed almost no cytotoxicity without X‐ray irradiation. In comparison, under 2 Gy or 8 Gy of X‐ray irradiation, cell viability of 4T1 cells dropped to 50% by increasing the Hf concentration of Hb@Hf‐Ce6 NPs up to 5.6 µg mL^−1^ (**Figure** **3**a). Clearly, the introduction of X‐ray radiation (8 Gy) could increase the cytotoxicity of the Hb@Hf‐Ce6 NPs to 4T1 cells. Therefore, the Hb@Hf‐Ce6 NPs can be exploited as a fantastic X‐ray radiosensitizer via X‐ray inducible RT‐RDT to exhibit a strong cytotoxicity to cancer cells. Furthermore, the X‐ray enhancement property of Hb@Hf‐Ce6 NPs was evaluated by clonogenic assay (Figure 3b,c). The percentages of 4T1 cell clones are shown in Figure 3b. The number of viable 4T1 cell colonies were counted, the lowest percentages of colonies can be found from Hb@Hf‐Ce6 NPs group with 8 Gy of X‐ray irradiation, giving evidence that the Hb@Hf‐Ce6 NPs can possess strong radiosensitizing effect.

Importantly, the high‐Z metal Hf in Hb@Hf‐Ce6 NPs can be used as a radiosensitizer to enhance RT‐RDT performance by ROS generation.^[^ ^18^ ^]^ Intracellular ROS production in 4T1 cells was detected via the 2,7‐dichlorofluorescin diacetate (DCFH‐DA) fluorescent probe. As showed in Figure 3d, the intracellular fluorescence intensities of dichlorodihydrofluorescein (DCF) in cells treated with Hb@Hf‐Ce6 NPs under X‐ray irradiation (NP+X‐ray) were obviously higher than other groups, indicating that Hb@Hf‐Ce6 NPs exhibited the best performance on RT‐RDT effectiveness by augmenting the ROS production. Moreover, DNA damage is the most lethal lesions induced by X‐ray.^[^ ^19^ ^]^ DNA damage extent was further measured by *γ*‐H2AX assay. Red fluorescence was found in the group with single X‐ray irradiation with a dose of 8 Gy. On contrast, strong fluorescence of *γ*‐H2AX can be observed from the Hb@Hf‐Ce6 NPs group with X‐ray irradiation, suggesting excellent RT‐RDT effect to lead DNA damage (Figure 3e). The enhanced RT‐RDT of Hb@Hf‐Ce6 NPs was further supported by Annexin V/PI apoptosis staining (Figure 3f,g). When cells treated with the Hb@Hf‐Ce6 NPs with 8 Gy of X‐ray irradiation, both early and late apoptosis ratios remarkably increased up to 77.0% compared with those of other groups. These results revealed that the Hb@Hf‐Ce6 NPs enhanced the anticancer effect of RT‐RDT by inducing cell apoptosis. Free radical reactions can lead to lipid peroxidation on cell membranes, which may render cellular dysfunction or death.^[^ ^20^ ^]^ Twofold higher level of lipid peroxidation was found in 4T1 cells treated with Hb@Hf‐Ce6 NPs under X‐ray irradiation in comparison with other groups (Figure 3h). It can be attributed to more ^1^O_2_ generation from Hb@Hf‐Ce6 NPs by RT‐RDT, leading to increased lipid oxidation of cell membranes. The above studies verified that the Hb@Hf‐Ce6 NPs mediated and ameliorated the anticancer biological effects by RT‐RDT. In addition, the penetration ability of Hb@Hf‐Ce6 NPs in solid tumors was evaluated using a 3D multicellular 4T1 cells spheroid model in vitro to mimic the dense structure on solid tumor (Figure S6, Supporting Information). Red fluorescence from Ce6 observed in middle slice of images, indicating Hb@Hf‐Ce6 NPs exhibited fabulous penetration property.

### 2.3 In Vivo Fluorescence Imaging and Biodistribution of Hb@Hf‐Ce6 NPs

**Figure 4:**
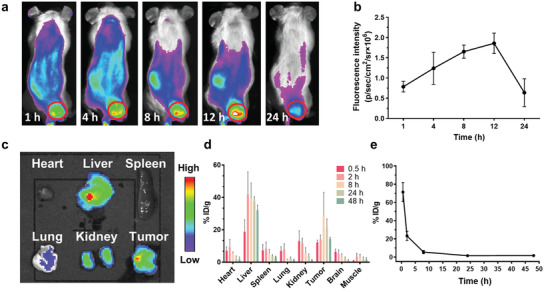
In vivo fluorescence imaging and biodistribution assay of Hb@Hf‐Ce6 NPs. a) The in vivo fluorescent images of 4T1 tumor‐bearing mice at 1, 4, 8, 12, and 24 h after injected with Hb@Hf‐Ce6 NPs. b) Quantification of the corresponding fluorescence intensity of mice at 1, 4, 8, 12, and 24 h. c) The ex vivo fluorescent images of main organs and tumors at 24 h. d) Biodistribution of Hb@Hf‐Ce6 NPs in various tissues after 0.5, 2, 8, 24, and 48 h intravenous administration. Hb@Hf‐Ce6 NPs concentrations were normalized as the percentage of the injected dose of hafnium per gram of each organ (%ID g^−1^). e) Blood circulation curve of Hb@Hf‐Ce6 NPs in mice through measuring the blood concentration of hafnium at 0.5, 2, 8, 24, and 48 h. Data are presented as mean values ± SD (*n* = 4).

Tumor accumulation and biodistribution of Hb@Hf‐Ce6 NPs were evaluated by in vivo fluorescent imaging. 4T1 tumor‐bearing Balb/c mice were injected intravenously with Hb@Hf‐Ce6 NPs. For in vivo fluorescence imaging, fluorescence signals of Ce6 from Hb@Hf‐Ce6 NPs can be rapidly found in tumor region at early time points and reach a plateau at 12 h post‐injection (**Figure** **4**a), suggesting the Hb@Hf‐Ce6 NPs could rapidly accumulate in tumors. Besides, fluorescence signals still existed at 24 h post‐injection. Quantitative analysis was conducted according to the fluorescence intensity (Figure 4b). Enhanced tumor accumulation of Hb@Hf‐Ce6 NPs benefited RT‐RDT. Mice were sacrificed for tissue collection after in vivo imaging. The ex vivo fluorescence imaging of the main organs and tumor indicated the high tumor accumulation of Hb@Hf‐Ce6 NPs (Figure 4c). These fluorescence imaging features endow Hb@Hf‐Ce6 NPs with the ability of tumor accumulation. To further investigate in vivo distribution of Hb@Hf‐Ce6 NPs, we measured the Hf concentration in different organs, tumors and bloodstream in mice after i.v. injection of Hb@Hf‐Ce6 NPs by ICP‐MS (Figure 4d). High amount of Hb@Hf‐Ce6 NPs can be found in tumor at various post‐injection timepoints, and the tumor accumulation reached a peak at 8 h post‐injection. Both fluorescence imaging and biodistribution results gave directly evidence that Hb@Hf‐Ce6 NPs could predominantly accumulate in the tumor. In this work, Ce6‐PEG‐polyphenols has been employed to enhance the blood circulation time of Hb@Hf‐Ce6 NPs.^[^ ^21^ ^]^ A further pharmacokinetic study was investigated in mice. Blood circulation curve and pharmacokinetic parameters of Hb@Hf‐Ce6 NPs were shown in Figure 4e and Table S1, Supporting Information. The half‐lives of biodistribution and elimination of Hb@Hf‐Ce6 NPs were about 1.01 and 19.31 h, respectively. The pharmacokinetic parameters were calculated based on the two‐compartment open model, indicating that Hb@Hf‐Ce6 NPs exhibited a long blood half‐life.

### 2.4 In Vivo Therapeutic Efficacy of Hb@Hf‐Ce6 NPs on an Orthotopic Combined Bilateral Tumor Model

**Figure 5:**
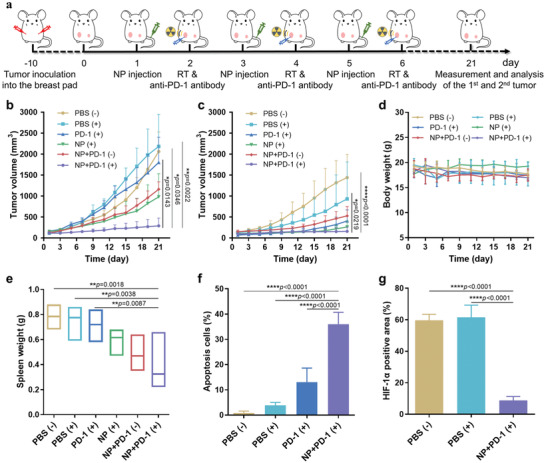
Hb@Hf‐Ce6 NPs mediated combination RT‐RDT and immunotherapy for 4T1 orthotopic and bilateral tumor model. a) Scheme illustrated the experimental steps for orthotopic and bilateral breast cancer mice model. Hb@Hf‐Ce6 NPs (NP, dose of Hf 6.15 mg kg^−1^) were intravenous injected into 4T1 tumor‐bearing mice on days 1, 3, and 5. On days 2, 4, and 6, the anti‐PD‐1 antibody (75 µg/mice) was injected locally in mice primary tumors combined with RT (6 Gy). Both sides of the tumors were measured and analyzed on day 21. b) Tumor volume growth curves for primary tumors. c) Tumor volume growth curves for distant tumors. d) The body weight of mice during treatment. e) The mice spleen weight on day 21 in different treatment groups. f) Immunofluorescence statistics of tumor slices on TUNEL assay. g) Quantitative analysis of HIF‐1*α* positive area. Data are presented as mean values ± SD (*n* = 4).

Encouraged by the promising tumor accumulation efficiency and long blood circulation time of Hb@Hf‐Ce6 NPs, we evaluated the anticancer efficacy in combination with immunotherapy in an orthotopic combined bilateral tumor model. The two‐tumor‐bearing mice were randomly divided into six groups when the primary tumor (left tumor) volume reached 100 mm^3^ (*n* = 4 in each group), including 1) untreated group, PBS (−); 2) the group treated with PBS plus X‐ray irradiation, PBS (+); 3) the group treated with anti‐PD‐1 antibody plus X‐ray irradiation, PD‐1 (+); 4) the group treated with Hb@Hf‐Ce6 NPs plus X‐ray irradiation, NP (+); 5) the group treated with Hb@Hf‐Ce6 NPs plus anti‐PD‐1 antibody, NP+PD‐1 (−); 6) the group treated with Hb@Hf‐Ce6 NPs plus anti‐PD‐1 antibody with X‐ray irradiation, NP+PD‐1 (+). A lower radiation dose of 6 Gy was chosen to reduce the toxicity and side effects of RT. The mice were treated for three times every 2 days and the treatment was monitored for 21 days (**Figure** **5**a). Both primary and distant tumor sizes were monitored with a digital caliper every other day (Figure 5b,c; Figures S7,S8, Supporting Information) and the tumors were collected and photographed on day 21 (Figures S9 and S10, Supporting Information). Compared with the control group, PBS (+) group has no obvious therapeutic effect. However, the growth rate of tumor volume in PBS (+) group was slower than that in the PBS (−) group, which slowed down the tumor growth to a certain extent. Moreover, the dose of RT used in this experimental animal model is low, so it is acceptable that the PBS (+) group does not have a good antitumor effect. In addition, it was found that treatment with Hb@Hf‐Ce6 NPs based X‐ray irradiation showed significant tumor growth inhibition effect on both primary and distant tumors, indicating a high antitumor efficacy (Figure 5b,c). Notably, treatment with combination of Hb@Hf‐Ce6 NPs based X‐ray irradiation and PD‐1 checkpoint blockade showed the highest tumor growth inhibition effect on both primary and distant tumors among all groups (Figure 5b,c; Figures S7,S8, Supporting Information). These results indicated that combination of Hb@Hf‐Ce6 NPs with RT‐RDT and PD‐1 immune checkpoint blockade induced efficient anticancer inhibition efficacy to eliminate the growth of both primary and distant tumors, promising for metastasis inhibition. The body weights of six groups of mice were also recorded every other day until day 21. As presented in Figure 5d, there's no big change in bodyweights among all treated mice. In addition, the weight of spleen after treatment was evaluated. The weight of PBS (−), PBS (+), PD‐1 (+), and NP (+) groups were much higher than NP+PD‐1 (−) and NP+PD‐1 (+) groups (Figure 5e). This result demonstrated that 4T1‐bearing mice exhibited the cancer associated splenomegaly, which is a typical symptom of mice with hyper‐enhancing metastatic tumors than any other non‐pulmonary‐metastatic tumor models.^[^ ^14^ ^]^ Additionally, monocytes formed in the enlarged spleen and migrated to the TME to promote immunosuppression.^[^ ^22^ ^]^ Besides, the spleen from mice of NP+PD‐1 (+) groups appeared in the normal morphology (Figure S11, Supporting Information). These results indicated that the combination treatment prevented spleen enlargement and alleviated immunosuppression. Owing to excellent performance if Hb@Hf‐Ce6 NPs in tumor inhibition, these X‐ray nanoprocessors were further used for tumor apoptosis and/or necrosis evaluation. TdT‐mediated dUTP nick end labeling (TUNEL) staining and hematoxylin and eosin (H&E) staining were performed (Figure 5f; Figures S12,S13, Supporting Information). In PD‐1 (+) group, a small percentage of TUNEL positive cells were observed in tumor slices due to anti‐PD‐1 antibody mediated immune effect. By contrast, high percentage of TUNEL positive cells (green fluorescence in images) were found in the tumor areas by the treatment of Hb@Hf‐Ce6 NPs combined with X‐ray irradiation and anti‐PD‐1 antibody, suggesting lots of tumor cells underwent apoptosis after treatments (Figure 5f; Figure S12, Supporting Information). These results were further confirmed by a majority of apoptosis or necrosis tumor cells found in tumors of NP+PD‐1 (+) group via H&E staining (Figure S13, Supporting Information).

It is known that hypoxia microenvironment existed in many types of tumors.^[^ ^23^ ^]^ The hypoxia microenvironment is associated with drug resistance and radioresistance.^[^ ^24^ ^]^ Overcoming the hypoxia stress is critical for RT. The as‐prepared Hb@Hf‐Ce6 NPs might release oxygen in situ for tumor hypoxia relief in vivo. Hence, a hypoxia‐inducible factor 1 (HIF‐*α*) probe was used for immunofluorescence examination (Figure 5g; Figure S14, Supporting Information). The tumor slice reflects the state of the entire tumor. In order to investigate the hypoxia in a wide range of tumor, images covering internal tumor and tumor boundary were provided to demonstrate hypoxia level of the tumor. The hypoxic area was stained by HIF‐*α* antibody (green). The green fluorescence in internal tumor (in rectangular box) was observed in PBS (−) and PBS (+) groups, whereas weak green fluorescence can be found in NP+PD‐1 (+) group, indicated that Hb@Hf‐Ce6 NPs can dramatically relieve hypoxia and improve the treatment effect under X‐ray irradiation. Additionally, blood chemistry analysis, including lactate dehydrogenase (LDH), creatine kinase (CK), alanine aminotransferase (ALT), aspartate transaminase (AST), blood urea nitrogen (BUN), and creatinine (CRE), was performed for systemic toxicity evaluation. All the parameters were in the normal range from all treatment groups, demonstrating the safety of the systemic administration of Hb@Hf‐Ce6 NPs with RT‐RDT and immunotherapy (Figure S15, Supporting Information). The above results were further confirmed by H&E‐stained tissue images. There was no pathological damage to the major organs of mice in all six groups (Figure S16, Supporting Information).

**Figure 6:**
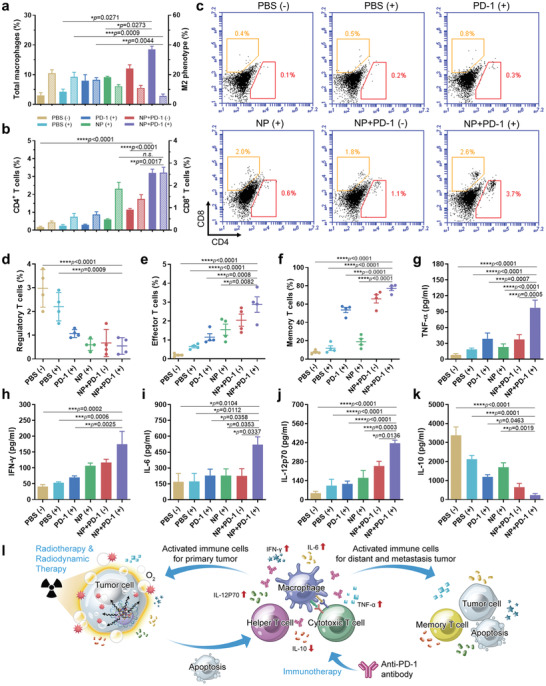
In vivo immunotherapy efficacy of Hb@Hf‐Ce6 NPs mediated RT‐RDT combined with anti‐PD‐1 antibody. a) Respective proportion of total macrophages (left column) and M2 phenotype macrophages (right column) in distant tumors. b) Respective proportion of CD4^+^ T cells (left column) and CD8^+^ T cells (right column) in distant tumors. c) Representative flow cytometry plots of CD4^+^ T cells and CD8^+^ T cells in distant tumors from six groups of mice. d) Respective proportion of regulatory T cells (CD3^+^, CD4^+^, Foxp3^+^) in distant tumors. e) Respective proportion of effector T cells (CD3^+^, CD8^+^, IFN*γ* ^+^) in distant tumors. f) Respective proportion of memory T cells (CD3^+^, CD8^+^, CD62L^low^, CD44^high^) in spleen. g–k) Cytokine levels of TNF‐*α*, IFN‐*γ*, IL‐6, IL‐12p70, and IL‐10 in serum from mice after various treatments. l) Schematic showed the potential mechanism of Hb@Hf‐Ce6 NPs mediated RT‐RDT‐Immunotherapy for immune response activation to inhibit both primary and distant tumors. Briefly, Hb@Hf‐Ce6 NPs accumulated at the tumor site through blood circulation. After X‐ray irradiation on the primary tumor, Hb@Hf‐Ce6 NPs can not only enhance the RT effect but also activate Ce6 with the assistance of radioluminescence by RDT to generate more ROS. The necrosis tumors in situ activated adaptive and innate immunity and caused a significant proinflammatory response. Furthermore, the anti‐PD‐1 antibody enhanced the immune response. Activated immunotherapy triggered both primary and distant tumor eradication and initiated the memory T cells generation to produce long‐term antitumor efficacy. Data are presented as mean values ± SD (*n* = 4).

In order to understand the potential immune mechanisms in relieving hypoxia activated by combined RT‐RDT‐immunotherapy with Hb@Hf‐Ce6 NPs, innate immune responses in both primary and distant tumors were assessed by flow cytometry on day 10 after three rounds of treatments on an orthotopic combined bilateral tumor model. Macrophages as antigen‐presenting cells are essential for inducing an innate immune response. Among them, M1‐phenotype and M2‐phenotype macrophages are two main extremes. M1 macrophages secrete pro‐inflammatory cytokines and chemokines which participate in positive immune response and immune surveillance. Otherwise, M2 macrophages have weak antigen‐presenting capabilities and play an important role in immune regulation by secreting inhibitory cytokines to downregulate the immune response.^[^ ^25^ ^]^ It was reported that tumor hypoxia directly promoted the polarization of macrophages into the immunosuppressive M2 phenotype.^[^ ^26^ ^]^ A hypoxia relief would be helpful for M1 macrophages dysregulated polarization. Thanks to oxygen delivery to the tumor sites by Hb@Hf‐Ce6 NPs, Hb@Hf‐Ce6 NPs plus PD‐1 (+) significantly induced higher frequency of total macrophages (CD11b^+^, F4/80^+^) and lower frequency of M2 macrophages (CD11b^+^, F4/80^+^, CD206^+^) infiltration in both primary and distant tumors when comparing to PBS control group (**Figure** **6**a; Figures S17,S18, Supporting Information). In addition, high percentage of M1 phenotype macrophages (CD86^+^) existed in both primary and distant tumors (Figures S19 and S20, Supporting Information). This result proved that Hb@Hf‐Ce6 NPs plus PD‐1 (+) combination therapy suppressed the polarization of immunosuppressive M2 phenotype caused by hypoxia, thereby promoting immune response.

Adaptive immunity is an immune response that can be identified and initiated against perinecrotic tumor cells, which exhibits the greatest advantage in specificity and durability.^[^ ^27^ ^]^ In our study, NP+PD‐1 (+) induced 3.20% CD4^+^ helper T cells and 2.58% CD8^+^ cytotoxic T lymphocytes infiltration in distant tumors, gated by CD3^+^ cells, which were 6.5‐fold and 7.4‐fold higher than PBS (−) group, respectively (Figure 6b,c; Figure S21, Supporting Information). Besides, these two kinds of T cells were also significantly infiltrated in primary tumors (Figures S22 and S23, Supporting Information). The enhanced distribution of CD8^+^ cells was verified by immunofluorescence images of distant tumor slices (Figure S24, Supporting Information). Furthermore, CD4^+^ T cells can be classified into two types, that is, helper T cells (CD3^+^, CD4^+^, Foxp3^−^), and regulatory T cells (CD3^+^, CD4^+^, Foxp3^+^). As mentioned previously, helper T cells can promote antitumor immune responses, while regulatory T cells have immunosuppressive effect. We tested the regulatory T cells in both distant and primary tumors. As provided in Figure 6d and Figure S25, Supporting Information, the percentage of regulatory T cells in NP+PD‐1 (+) group was greatly reduced to less than 1% in distant tumors and less than 0.5% in primary tumors, which indicates that Hb@Hf‐Ce6 NPs mediated by anti‐PD‐1 antibody were able to effectively abrogate the activity of regulatory T cells. In addition, group of Hb@Hf‐Ce6 NPs+PD‐1 (+) was most efficient to induce the conversion of naive T cells to effector T cells (CD3^+^, CD8^+^, IFN‐*γ* ^+^) in both distant and primary tumors (Figure 6e; Figure S26, Supporting Information). These results demonstrated that the cold‐tumors were activated to hot‐tumors because of the adaptive immune activation by X‐ray irradiation triggered RT‐RDT and PD‐1 checkpoint blockade. These results strongly suggested that Hb@Hf‐Ce6 NPs (+) plus anti‐PD‐1 antibody not only induced innate immune response but also augmented the tumor‐specific adaptive response in tumors.

To investigate the long‐term antitumor immune memory induced by the combination treatment, memory T cells (CD3^+^, CD8^+^, CD44^high^, CD62L^low^) in the splenocytes were harvested and analyzed in different groups of mice (Figure S27, Supporting Information; Figure 6f). Memory T cells induced a strong immune memory protection effect in the combination therapy group and increased by an ≈14.6‐fold compared to the PBS (−) group. This synergistic combination elicited systemic antitumor immunity by boosting long‐term immune response. Antitumor immune response by the combination treatment of RT‐RDT with PD‐1 checkpoint blockade was further determined by measuring cytokine secretion via enzyme linked immunosorbent assay (ELISA). After treatment, serum of mice from all groups was collected and five kinds of specifically related cytokines, including tumor necrosis factor‐*α* (TNF‐*α*), interferon‐*γ* (IFN‐*γ*), interleukin‐6 (IL‐6), interleukin‐12 (IL‐12), and interleukin‐10 (IL‐10) were tested (Figure 6g–k). The combination RT‐RDT and anti‐PD‐1 therapies induced 11.9‐, 4.3‐, 3.1‐, and 5.9‐fold higher secretion of TNF‐*α*, IFN‐*γ*, IL‐6, and IL‐12 than those of PBS (−) group, respectively. 14.1‐fold lower secretion of IL‐10 was found in the combination treatment group, indicating RT‐RDT cooperated with anti‐PD‐1 antibody greatly caused a significant proinflammatory response. In summary, Hb@Hf‐Ce6 NPs were used to enhance RT‐RDT immunotherapy for both primary and distant tumors through reactivation of immune cells and cytokines release that maintained and promoted immune cells proliferation. In addition, the PD‐1 checkpoint blockade solved the issues of low immune response rate caused by RT‐RDT, which led to an abscopal and long‐term therapeutic efficacy (Figure 6l).

### 2.5 In Vivo Therapeutic Efficacy of Hb@Hf‐Ce6 NPs on Lung Metastasis Model

**Figure 7:**
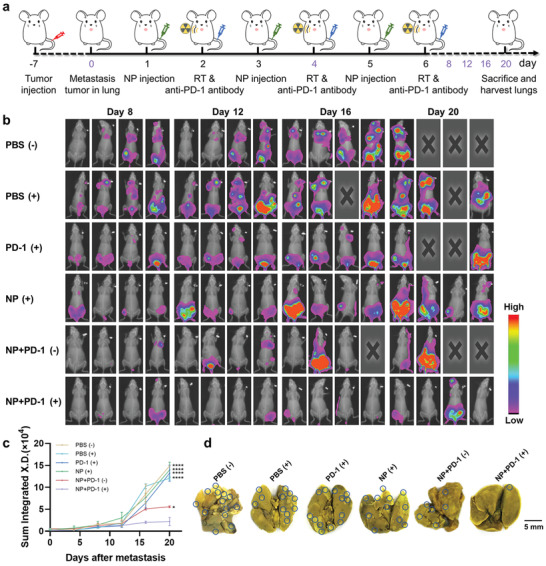
Hb@Hf‐Ce6 NPs mediated combination RT‐RDT and immunotherapy for the 4T1 tumor metastasis model. a) Scheme illustrated the experimental steps for 4T1 tumor metastasis model. 4T1‐luc cells were intravenous injected into female mice on day ‐7. Hb@Hf‐Ce6 NPs (dose of Hf 6.15 mg kg^−1^) were injected into mice on days 1, 3, and 5. On days 2, 4, and 6, the anti‐PD‐1 antibody (75 µg) was injected intravenously in mice with lung RT (6 Gy). Bioluminescence images of mice were taken on days 0, 4, 8, 12, 16, and 20. Mice were sacrificed on day 20 and lungs were obtained for analysis. b,c) Respective bioluminescence images (on days 8, 12, 16, and 20) and signals of mice bearing 4T1‐luc cells showed the development of metastasis. d) Representative photos showed the gross appearance of tumor in lungs. Data are presented as mean values ± SD (*n* = 4). *p < 0.05; ****p < 0.0001.

In order to further evaluate the efficacy of Hb@Hf‐Ce6 NPs for enhanced combination therapy against tumor metastasis, a lung metastasis model on Balb/c female mice was conducted. This model is innovative to mimic clinical treatment and reflect the therapeutic efficacy of Hb@Hf‐Ce6 NPs combined with RT‐RDT immunotherapy intuitively. 2 × 10^6^ firefly luciferase 4T1 (4T1‐luc) cells were intravenously injected into the mice, and 7 days later, 4T1‐luc tumors can be detected by bioluminescence in the lungs of mice by intraperitoneal injection of D‐luciferin, a substrate of luciferase (Figure S28, Supporting Information). All the mice were divided into six groups randomly as previous model. The tumor metastasis was monitored on days 0, 4, 8, 12, 16, and 20 by bioluminescence imaging (**Figure** **7**a). Bioluminescence images and corresponding florescence intensity in different treatment of mice were shown in Figure 7b,c. Early metastasis was found in the lung on day 0 (Figure S28, supporting information). However, on day 8, stronger fluorescence signals of 4T1 were found in other tissues such as breast pad and abdomen (Figure 7b). These results demonstrated that tumor metastasis can be observed in the lung at the early stage and spread throughout the body in the late stage.

Besides, three mice died in PBS (+) on day 20. In addition, large‐area visible metastasis displayed in the group of PBS (+), PD‐1 (+), and NP (+). In remarkable contrast, the group of NP+PD‐1 (+) exhibited delayed metastasis without individual mouse death, showing effective treatment efficacy of the combination strategy of RT‐PDT and PD‐1 checkpoint blockade immunotherapy based on the Hb@Hf‐Ce6 NPs. Lung metastasis inhibition was further analyzed by counting the metastasis nodes of lungs on the day 20. Obviously, lowest number of metastasis nodes were found in lungs of NP+PD‐1 (+) group among all six groups (Figure 7d). It demonstrated that combinational RT‐PDT and immunotherapy by Hb@Hf‐Ce6 NPs could suppress lung colonization of tumor cells much more efficiently than RT‐PDT and PD‐1 checkpoint blockade therapy separately.

## 3 Discussion

RT as the pillar of tumor treatment has shown tremendous promises for dealing with a range of aggressive tumors by excellent penetration depth of tissues. RT application is hindered by critical issues including dose‐dependent toxicity, weak therapeutic effects for large tumors and tumor metastasis, low sensitivity in hypoxic TME.^[^ ^28^ ^]^ To solve these problems, considerable efforts have been devoted to multifunctional radiosensitizers.^[^ ^29^ ^]^ Radiosensitizers make tumors with admirable radiosensitivity, and accumulate X‐ray at the tumor to improve the RT effectiveness.^[^ ^30^ ^]^ In our developed nanoprocessor Hb@Hf‐Ce6 NPs, Hf serves as a dual functional radiosensitizer by increasing absorption of radiation energy to produce additional low‐energy electrons and transferring absorbed X‐ray energy to Ce6 to boost high level ROS based on the Hf‐polyphenol coordination platform. In that case, high level of ROS generation by RT‐RDT induces gene damage and promotes lipid oxidation for cancer cells death.

As immunosuppressive TME is to do with hypoxia stress, oxygen enrichment in tumor would relieve the hypoxia environment and radioresistance to enhance RT efficacy.^[^ ^31^ ^]^ Herein, Hb@Hf‐Ce6 NPs can also be employed as an oxygen transporter, which directly delivers abundant oxygen into the tumor site. Oxygen perfusion by Hb@Hf‐Ce6 NPs exhibits these following advantages: 1) alleviating tumor hypoxia state; 2) greatly enhancing the RT effectiveness and overcoming radioresistance; and 3) acting as an oxygen donor for achievement of RDT by Ce6. Thus Hb@Hf‐Ce6 NPs can break through intrinsic immunosuppressive TME to modulate macrophage polarization.

It is known that the lack of T‐cell infiltration in TME impairs the therapeutic effect of PD‐1 checkpoint blockade.^[^ ^32^ ^]^ Particularly, RT can also enhance checkpoint blockade immunity.^[^ ^33^ ^]^ In our work, this augmented RT‐RDT and PD‐1 checkpoint blockade combinatory X‐ray nanoprocessor platform greatly improves cancer therapeutic outcomes, resulting in several antitumor immunities. First, high level ROS by RT‐RDT leads to cell apoptosis in primary tumor, which might increase effector T cells infiltration. Second, this combination approach extends a synergistic effect to distant tumor. A number of immune cells (e.g., helper T cells and effector T cells) are recruited to distant tumors favoring effective cancer inhibition, thereby secreting proinflammatory cytokines such as TNF‐*α*, IFN‐*γ*, IL‐12P70 to inhibit cancer development and progression. Third, this combination formula reduces immune evasion‐related regulatory T cells and increases generation of memory T cells for abscopal antitumor effect. Furthermore, this combination strategy achieves remarkable tumor metastasis inhibition.

## 4 Conclusion

In summary, we develop a novel and versatile strategy to carry Hb based on the self‐assembly coordination of metals and Ce6 modified polyphenols (Hb@Hf‐Ce6 NPs). As an efficient metal‐phenolic X‐ray nanoprocessor, our Hf based X‐ray nanosensitizers combine Hb oxygen delivery and RT together, greatly overcome the resistance of RT and improve RT‐PDT efficacy. These Hb@Hf‐Ce6 NPs possess tumor‐homing ability with a long blood circulation time. Moreover, the nanoplatform can modulate hypoxic TME to reverse immunosuppression, which takes advantage of oxygen transport by Hb@Hf‐Ce6 NPs. The synergistic effect of RT‐PDT and PD‐1 checkpoint blockade effectively elicits superior antitumor efficacy on bilateral tumor model and lung metastatic model of 4T1 breast cancer. This combinatory formula not only offers an excellent therapeutic effect of primary tumor but also exerts a remote antitumor effect to distant tumor by T cells mediated systemic immunities. With good biocapacity and safety, our fabricated Hb@Hf‐Ce6 NPs based theranostic nanoplatform shows promising potential in clinical application for effective RT‐RDT in combination with checkpoint blockade immunotherapy.

## 5 Experimental Section

##### Preparation of Ce6‐PEG‐Polyphenols

The Ce6‐modified PEG polyphenol was prepared following a reported protocol with some modification.^[^ ^17^ ^]^ 8‐arm PEG‐NHS (20 kDa, 500 mg) and chlorin e6 monolysine amide trisodium salt (7.5 mg) were added in DMF (4 mL) individually and mixed in argon for 20 min. Then dopamine hydrochloride (190 mg) was added in DMF and mixed with above solution for 1 h. Later, TEA (95 µL) was added into the above solution under argon protection overnight. On the second day, glycine (50 mmol L^−1^ pH = 3) solution was added into the above solution, and purified by dialysis for two days, followed by lyophilization.

##### Preparation of Hb@Hf‐Ce6 NPs

First, Hb water solution was added iron powder and stirred for 3 days. The iron powder was removed from the Hb solution by centrifuge (3000 rpm, 4 min). Hb (150 µL, 5 mg mL^−1^), HfCl_4_ (50 µL, 3 mg mL^−1^), GA (300 µL, 5 mg mL^−1^), Ce6‐PEG‐polyphenols (75 µL, 150 mg mL^−1^), and 1 mL milli‐Q water were mixed and stirred for 3 h. Afterward, the Hb@Hf‐Ce6 NPs were purified by dialyzing against water for three times to remove the free ions. Finally, Hb@Hf‐Ce6 NPs were obtained after ultrasound for 10 min.

##### Cell Viability Assay

The murine mammary carcinoma 4T1 cells were cultured in 96‐well plate at the density of 4000/well for 24 h. Hb@Hf‐Ce6 NPs were added to the cells by different dosages of Hf and incubated for other 6 h. Then, 4T1 cells were exposed to X‐ray (2 Gy and 8 Gy) and changed to fresh 1640 medium. After further incubation of 4T1 cells for 48 h, the relative cell viabilities were measured and calculated. Cell viability (%) = (OD_490 sample_ – OD_490 blank_) / (OD_490 control_ – OD_490 blank_) × 100%.

##### Clonogenic Assay

The radio enhancement property of Hb@Hf‐Ce6 NPs was also evaluated by clonogenic assay including images and statistics. 4T1 cells were prepared in 6‐well plate at 1000/well and then incubated for 24 h. Cells were treated with/without Hb@Hf‐Ce6 NPs for 6 h followed by irradiation (8 Gy). After 6 days of routine incubation, 4T1 cells were fixed with 4% paraformaldehyde (PFA), then stained with crystal violet to count the cloning cluster. Surviving fraction = (surviving colonies) / (cells seeded × plating efficiency). Plating efficiency = number of colonies counted / number of cells seeded.

##### DCFH‐DA Assay

2′,7′‐dichlorofluorescin diacetate (DCFH‐DA) was used to monitor intercellular ROS production. First, 4T1 cells were seeded in confocal dishes overnight. Then, the cells were treated with/without Hb@Hf‐Ce6 NPs for 6 h followed by washed three times and stained with 30 µm DCFH‐DA probe. After that, X‐ray (8 Gy) was utilized for irradiation. Finally, the 4T1 cells were washed three times and imaged by confocal laser scanning microscopy (CLSM Nikon A1R).

##### DNA Damage

The *γ*‐H2AX experiment was performed to detect DNA breaks.^[^ ^34^ ^]^ HCS DNA Damage Kit (Invitrogen) was used to determine DNA damage. Briefly, 4T1 cells were first cultured on confocal dishes overnight. The next day the cells were incubated with Hb@Hf‐Ce6 NPs for 6 h and irradiated with X‐ray (8 Gy). After 24 h, the 4T1 cells were washed by PBS and fixed by 4% PFA solution for 15 min. After washing by PBS for three times, the cells were permeabilized by Triton‐100 solution. Afterward, the cells were exposed to BSA blocking buffer and then further incubated with pH2AX mouse monoclonal antibody for 2 h. 4T1 cells were stained by Alexa Fluor 555 goat anti‐mouse IgG (dilution of 1:2000) and Hoechst 33342 (dilution of 1:6000) for 1 h at room temperature. Finally, 1 mL PBS was added to each dish and proceed to imaging by CLSM (Nikon A1R).

##### Cell Apoptosis Assay

Cell apoptosis was evaluated by FITC Annexin V Apoptosis Detection Kit (BD Biosciences). Following the protocol, 4T1 cells were cultured in 6‐well with/without Hb@Hf‐Ce6 NPs, and then two groups (X‐ray and NP+X‐ray) irradiated with X‐ray (8 Gy). After 24 h, cells were resuspended in binding buffer (100 µL) and co‐cultured with Annexin V‐FITC and propidium iodide mixed solution sheltered from the light for 15 min. Last, gently vortexed the 4T1 cells, incubated for 15 min and added the binding buffer for analyzing via flow cytometry (Beckman Cytoflex S flow cytometer).

##### Malondialdehyde (MDA) Assay

Lipid peroxidation levels of X‐PDT were evaluated by MDA assay kit. Briefly, 4T1 cells were seeded in 6‐well plate with/without Hb@Hf‐Ce6 NPs for 6 h. Two groups (X‐ray and NP+X‐ray) were irradiated by X‐ray (8 Gy). Finally, 24 h later the cells were harvested, and lipid peroxidation levels were measured using MDA Assay Kit following the protocol (Beyotime Biotechnology, China).

##### In Vitro Penetration in 3D Multicellular Tumor Spheroids

To evaluate the Hb@Hf‐Ce6 NPs penetration, a 3D multicellular 4T1 cells spheroid model was prepared. Corning spheroid microplates were used to construct multicellular spheroid models. First, 4T1 cells were seeded in plates at 500/well for 5 days to yield multicellular spheroids. The culture medium was replaced by Hb@Hf‐Ce6 NPs and co‐cultured for another 24 h. And then, the multicellular spheroids were fixed by 4% PFA for 15 min followed by wash with PBS. Later, the tumor spheroids stained by DAPI for 15 min. Finally, multicellular spheroids were observed by CLSM (Nikon A1R).

##### Animal Model

The animal experiment procedures were conducted following an approved protocol (UMARE‐030‐2018) by the University of Macau Animal Ethics Committee.

##### In Vivo Fluorescence Imaging

For the in vivo fluorescence imaging experiment, 4T1 tumor‐bearing mice were prepared for imaging. When the 4T1 tumor volume reached around 100 mm^3^, Hb@Hf‐Ce6 NPs solution was intravenously injected into the mice. IVIS LuminaXR was used to scan the whole mice body at time points (1, 4, 8, 12, and 24 h). The data was calculated by region of interest.

##### Biodistribution of Hb@Hf‐Ce6 NPs

4T1 cells (2 × 10^6^) were used to inoculate a subcutaneous tumor on the right flank of each Balb/c mice. After 6 days, the tumor volume was about 100 mm^2^ (6–8 mm). Mice were randomly divided into five groups (0.5, 2, 8, 24, 48 h). Mice were injected with Hb@Hf‐Ce6 NPs (the dose of Hf is 100 µg/mice) intravenously. Blood, heart, liver, spleen, kidney, lung, tumor, brain, and muscle were collected and weighed. All the organs were mixed with 500 µL milli‐Q water by a homogenizer mixer. Blood was collected with 200 µL heparin sodium (5 mg mL^−1^) and 100 µL of the mixed solution was used for digestion. All the organs and blood were digested with 0.4 mL H_2_O_2_ and 1.6 mL 63% nitric acid for 3 days. The solutions were filtered and the volume was made constant in 10 mL with 2% nitric acid. Hf concentration in tissues was determined by ICP‐MS and analyzed by standard curve. %ID g^−1^ = Hf_tissue lysate_ × *V*_tissue lysate_ / (Hf_injected_ × *V*_injected Hf_ × tissue weight) × 100%.

##### In Vivo 4T1 Orthotopic and Bilateral Tumor Model

Six‐week‐old female Balb/c mice were obtained for in vivo study. Both sides of the breast pads in mice were injected with 4T1 cells to form primary tumor (1 × 10^6^/mice) and distant tumor (5 × 10^5^/mice). Therefore, orthotopic and bilateral breast cancer mice model were established. 24 mice were divided into six groups (4 mice per group): PBS (−) for PBS; PBS (+) for PBS + X‐ray; PD‐1 (+) for anti‐PD‐1 antibody + X‐ray; NP (+) for Hb@Hf‐Ce6 NPs + X‐ray; NP+PD‐1 (‐) for Hb@Hf‐Ce6 NPs + anti‐PD‐1 antibody; NP+PD‐1 (+) for Hb@Hf‐Ce6 NPs + anti‐PD‐1 antibody + X‐ray. The doses of Hf, anti‐PD‐1 antibody, and X‐ray were 6.15 mg kg^−1^, 75 µg/mice, and 6 Gy at a time, respectively. Only the primary tumor received X‐ray irradiation and all the other part of mice was shielded by lead cover. Following the schematic illustration treatment method, the tumor sizes were calculated every other day according to the following formula: width^2^ × length / 2. Mice spleens were also collected and weighed.

##### Flow Cytometry Analysis

The immune cells were analyzed following the standard protocol. Briefly, tumors were collected and incubated in dissociation buffer with 1640 medium (contained collagenase, hyaluronidase, and deoxyribonuclease I) at 37 °C for digest tumor tissues to the single‐cell suspension. And then the cells were stained with surface antibodies: CD3‐PE (eBioscience, Catalog: CD0304), CD4‐FITC (eBioscience, Catalog: 11‐0041‐82), CD8‐PerCP‐Cy5.5 (eBioscience, Catalog: 45‐0081‐82), CD86‐PE (eBioscience, Catalog: 12‐0862‐81), CD11b‐FITC (eBioscience, Catalog: 11‐0112‐81), F4/80‐PE‐Cy5 (eBioscience, Catalog: 15‐4801‐82), CD206‐PE (eBioscience, Catalog: 12‐2061‐82), CD44‐PE (eBioscience, Catalog: 12‐0441‐81), or CD62L‐APC (eBioscience, Catalog: 17‐0621‐81) respectively for 30 min. After fixing and perforating the cells, the intracellular markers: FOXP3‐PE (eBioscience, Catalog: 12‐5773‐82) or IFN‐*γ*‐PE (eBioscience, Catalog: 12‐7311‐82) were stained. The stained cells were detected using flow cytometry (Beckman Cytoflex S flow cytometer). The data were analyzed using FlowJo 10.0.

##### Enzyme‐Linked Immunosorbent Assay

The expression level of cytokines in the serum of mice after 21 days with different treatments including IFN‐*γ*, TNF‐*α*, IL‐6, IL‐10, and IL‐12p70 were detected by commercial ELISA kits (Neobioscience Biotechnology, Shenzhen, China) following the protocol.

##### Biochemistry Index Analysis

Serum of mice after 21 days with different treatments was collected to test the biomedical parameters including lactate dehydrogenase (LDH), creatine kinase (CK), alanine aminotransferase (ALT), aspartate transaminase (AST), blood urea nitrogen (BUN), and creatinine (CRE), which were measured by the assay kits (Nanjing Jiancheng Bioengineering Institute, China).

##### Immunofluorescence Staining

Tumor tissues were cut for ten‐micrometer sections by using frozen tissue sectioning (Leica CM5030 Cryostat). Tumor tissue slices were collected and incubated with CD3, CD8, or HIF‐*α* antibodies overnight at 4 °C. And then, the samples were stained with DAPI after washing with PBS in three times. The tissue sections were observed under CLSM (Nikon A1R Confocal System).

##### TUNEL Assay

First, the distant tumor tissues were obtained and cut by using frozen tissue sectioning. And then, the sections were prepared according to the standard protocol provided for the TUNEL assay by Andy Fluor 488 Apoptosis Detection Kit (Wuhan ABP‐Biosciences Co., Ltd).

##### Hematoxylin and Eosin (H&E) Staining

The heart, liver, spleen, kidney, lung, and distant tumor tissues were obtained and fixed with 4% PFA. And then the H&E stained samples were prepared by Shuen Biotechnology, Shanghai. The photos of tissue sections were obtained by a CLSM (Nikon A1R Confocal System).

##### In Vivo 4T1 Tumor Metastasis Model

Six‐week‐old female Balb/c mice were used for in vivo 4T1 tumor metastasis model. 2 × 10^6^ 4T1‐luc cells were implanted in each mouse intravenously. 7 days later, 4T1 tumor metastasis model in lung was established. 24 mice were divided into six groups (4 mice per group): PBS (−) for PBS; PBS (+) for PBS + X‐ray; PD‐1 (+) for anti‐PD‐1 antibody + X‐ray; NP (+) for Hb@Hf‐Ce6 NPs + X‐ray; NP+PD‐1 (‐) for Hb@Hf‐Ce6 NPs + anti‐PD‐1 antibody; NP+PD‐1 (+) for Hb@Hf‐Ce6 NPs + anti‐PD‐1 antibody + X‐ray. The doses of Hf, anti‐PD‐1 antibody, and X‐ray were 6.15 mg kg^−1^, 75 µg/mice, and 6 Gy at a time, respectively. Following the schematic illustration treatment method, Hb@Hf‐Ce6 NPs were injected on days 1, 3, and 5. Only the lungs were irradiated by X‐ray locally, and the anti‐PD‐1 antibody was administered intravenously. Both the X‐ray irradiation and anti‐PD‐1 antibody were performed on days 2, 4, and 6. The tumor bioluminescence imaging was observed on days 0, 4, 8, 12, 16, and 20 by in vivo extreme system (Bruker). On the day 20, the representative lungs of each group of mice were removed, fixed by Bouin's solution and washed by 70% ethanol for imaging.

##### Statistical Analysis

All data were presented as mean ± SD. Statistical differences were performed using the ordinary one‐way ANOVA analysis, followed by Dunnett's multiple comparisons test of the GraphPad Prism 8.0.2 (GraphPad Software, Inc., USA). Statistical significance was assigned at *p < 0.05, **p < 0.01, ***p < 0.001, and ****p < 0.0001.

## Conflict of Interest

The authors declare no conflict of interest.

## Supporting information

Supporting Information
