# Supplementary material for: Oxygen‐Enriched Metal‐Phenolic X‐Ray Nanoprocessor for Cancer Radio‐Radiodynamic Therapy in Combination with Checkpoint Blockade Immunotherapy
Source: Adv Sci (Weinh). 2020 Dec 31;8(4):2003338. doi: 10.1002/advs.202003338 (PMC7887592; doi:10.1002/advs.202003338)
Supplement: Supplementary file 1 — Supporting Information [file ADVS-8-2003338-s001.pdf]

© 2020 Wiley-VCH GmbH

ADVANCED SCIENCE
Open Access

Supporting Information

for *Adv. Sci.,* DOI: 10.1002/advs.202003338

Oxygen Enriched Metal-Phenolic X-Ray Nanoprocessor for Cancer Radio-Radiodynamic Therapy in Combination with Checkpoint Blockade Immunotherapy

Wei Sang†, Lisi Xie†, Guohao Wang, Jie Li, Zhan Zhang, Bei Li, Sen Guo, Chu-Xia Deng, Yunlu Dai*

Supporting Information

Oxygen Enriched Metal-Phenolic X-Ray Nanoprocessor for Cancer Radio-Radiodynamic Therapy in Combination with Checkpoint Blockade Immunotherapy

*Wei Sang†, Lisi Xie†, Guohao Wang, Jie Li, Zhan Zhang, Bei Li, Sen Guo, Chu-Xia Deng, Yunlu Dai**

W. Sang, Dr. L. Xie, G. Wang, Dr. J. Li, Z. Zhang, Dr. B. Li, S. Guo, Prof. C.-X. Deng and Prof. Y. Dai
Cancer Center, Faculty of Health Sciences
University of Macau
Macau SAR, 999078, China
*Email: yldai@um.edu.mo

W. Sang, Dr. L. Xie, G. Wang, Dr. J. Li, Z. Zhang, Dr. B. Li, S. Guo, Prof. C.-X. Deng and Prof. Y. Dai
Institute of Translational Medicine, Faculty of Health Sciences
University of Macau
Macau, SAR, 999078, China

†W. Sang and L. Xie contributed equally to this work.

Materials: Triethylamine (TEA), N,N-dimethylformamide, anhydrous (DMF), 3-(4,5-dimethylthiazol-2-yl)-2,5-diphenyltetrazolium bromide (MTT), iron (III) chloride ($FeCl_3$), and 5-hydroxydopamine hydrochloride (98%) were purchased from Sigma-Aldrich. Phosphate buffered saline (PBS) and RPMI-1640 medium were purchased from Thermo Fisher Scientific. Annexin V/PI apoptosis detection kit was purchased from BD Biosciences. 8-arm PEG Succinimidyl Glutarate (tripentaerythritol) was purchased from JenKem Technology USA. Chlorin e6 monolysine amide trisodium salt were purchased from Santa Cruz Biotechnology, Inc.

Characterization: UV-Vis absorption spectra were obtained on a SHIMADZU UV-1800 spectrophotometer. Proton nuclear magnetic resonance (^{1}H-NMR) spectra were recorded on the Bruker Ascend 400 MHz spectrometers using dimethyl sulfoxide-d_6 as the solvent. Transmission electron microscopy (TEM) images were acquired on a JEM-3010UHR/JEM-2100F field emission electron microscope. Energy dispersive X-ray spectroscopy (EDS) analysis were measured by Zeiss Sigma. The concentration of Hf were detected by the iCAP^{TM} Q inductively coupled plasma mass spectrometry (ICP-MS). The particle sizes of Hb@Hf-Ce6 NPs were determined by dynamic light scattering (Malvern Zetasizer Nano ZSP system ZEN5600). The oxygen concentration in water was monitored using an Ysi Dissolved Oxygen Meter. O_2 release was measured by the $Ru(bpy)_3Cl_2$ probe through fluorescence spectrophotometer (FluoroMax-4, Horiba, Japan). Briefly, pure oxygen was poured into 500 μl aqueous solution of Hb@Hf-Ce6 NPs (425 μg mL^{-1} of Hb) 5 min. And then the Hb@Hf-Ce6 NPs were irradiated with X-ray (5 mGy, 10 min). $Ru(bpy)_3Cl_2$ (0.1mol L^{-1}) 10 μl was added into above solution rapidly, and finally the fluorescence intensity from $Ru(bpy)_3Cl_2$ was acquired by fluorescence spectroscopy (600 nm) at predetermined time points. The ratio of fluorescence intensity was obtained from $Ru(bpy)_3Cl_2$ before reaction (I_0) and after reaction (I_n). $^1O_2$ generation was detected by SOSG assay kit. Hb@Hf-Ce6 NPs, H_2O and Ce6 with/without

X-ray irradiation contained 1 μM SOSG. After incubating for 30 min, the solutions were detected by green fluorescence, respectively.

Cell line and animal: The 4T1 murine mammary carcinoma cell line was purchased from the American Type Culture Collection. The cells were cultured in complete RPMI 1640 medium containing 10% fetal bovine serums (FBS), penicillin (100 U mL^{-1}), and streptomycin (100 μg mL^{-1}; Gibco, USA). 4T1 cells were cultured in 37°C incubator with humidified atmosphere of 5% CO_2 and subcultured by 0.05% trypsin-EDTA dissociation reagent. Six-week-old female Balb/c mice were obtained from Animal Research Core of Faculty of Health Sciences, University of Macau.

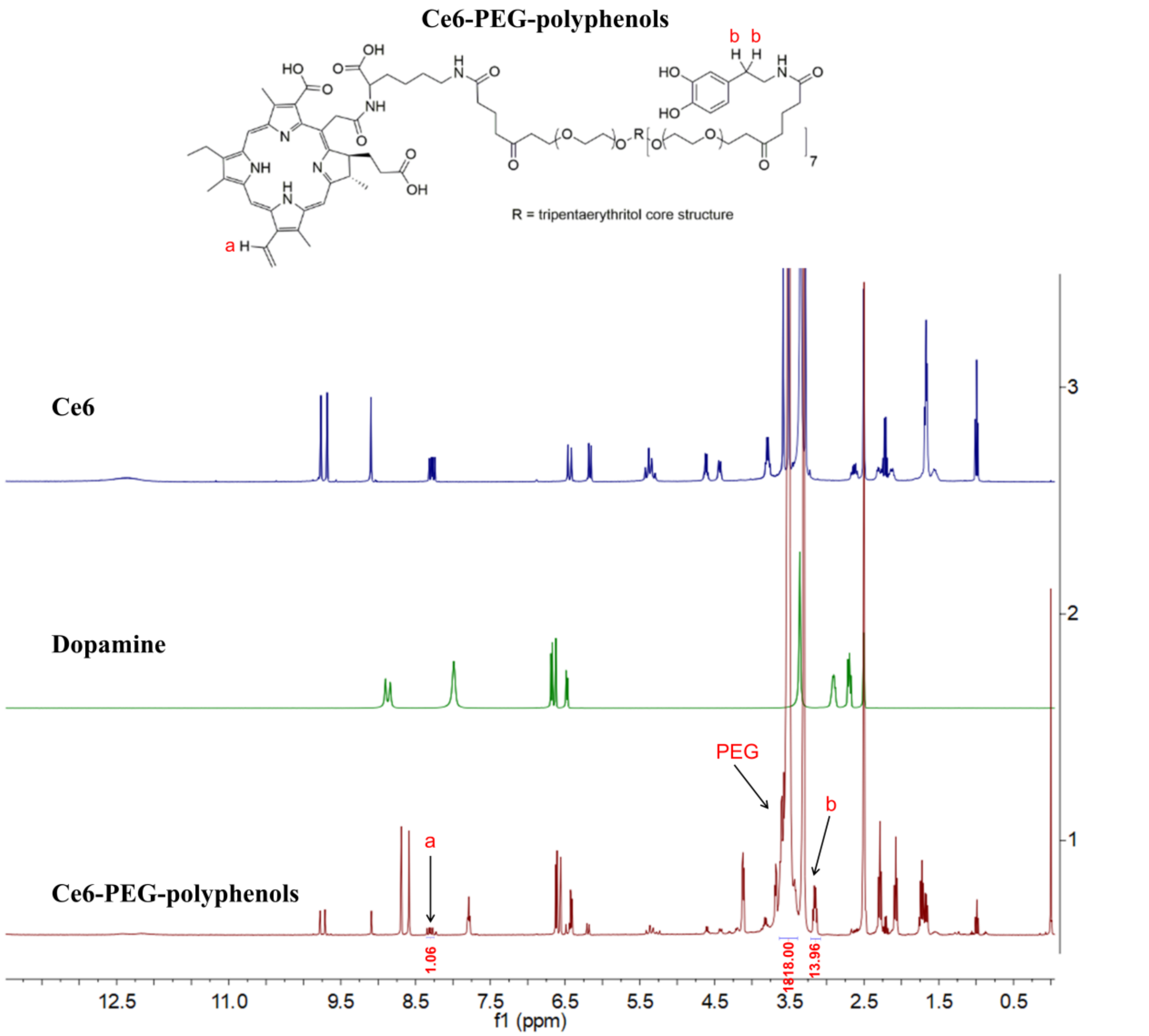

Figure S1. The ^{1}H NMR spectrum (400 MHz, DMSO-d_6, room temperature) of dopamine, Ce6 and Ce6-PEG-polyphenols.

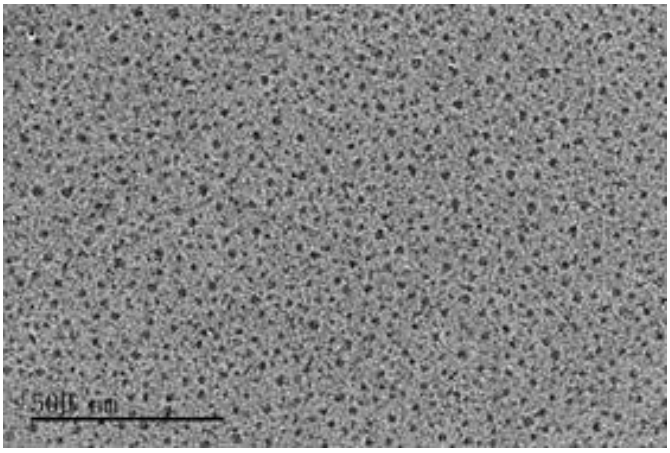

Figure S2. TEM image of Hb@Hf-Ce6 NPs. Scale bar: 500 nm.

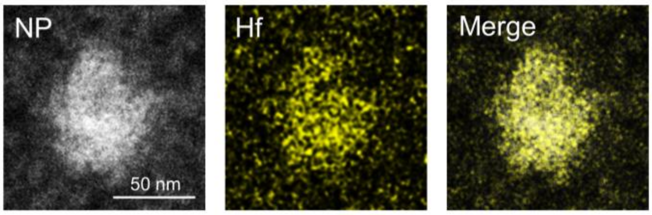

Figure S3. High-angle annular dark-field scanning transmission electron microscopy (HAADF-STEM) with energy-dispersive X-ray spectroscopy (EDX) mapping of Hb@Hf-Ce6 NPs.

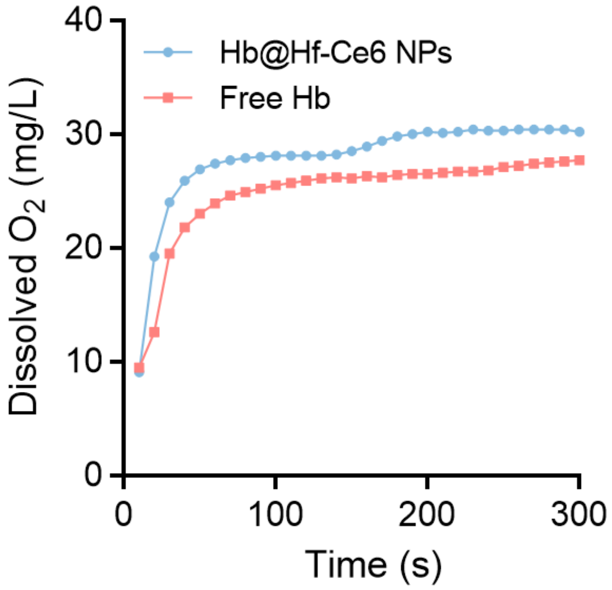

Figure S4. Oxygen dissociation curves of free Hb and Hb@Hf-Ce6 NPs.

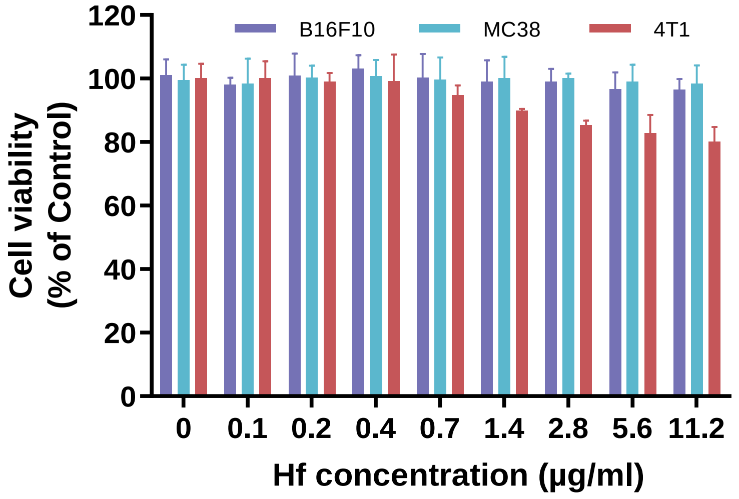

Figure S5. The cell viability treated with different concentrations of Hb@Hf-Ce6 NPs on B16F10, MC38 and 4T1 cells. Data are presented as mean values ± SD (n=5).

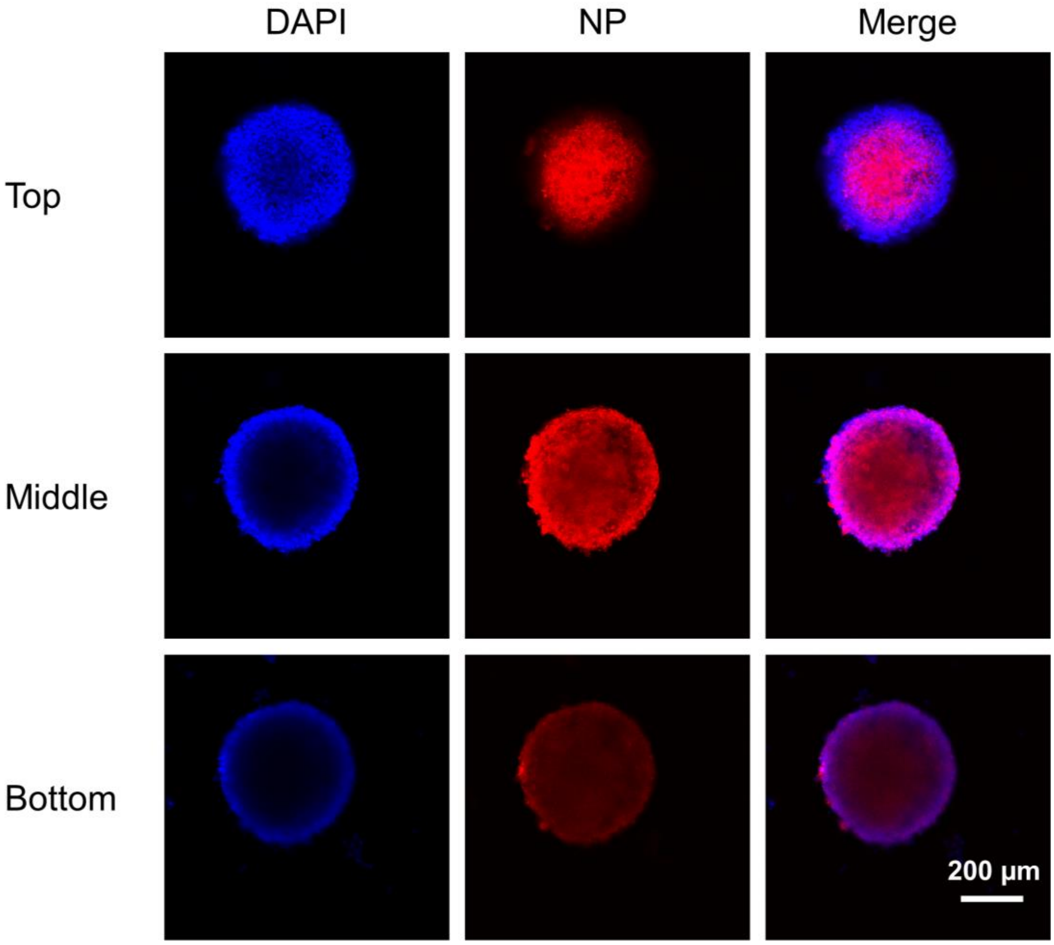

Figure S6. Penetration of Hb@Hf-Ce6 NPs in 4T1 multicellular tumor spheroids. Representative confocal microscopy images from various layers. Scale bar: 200 μm.

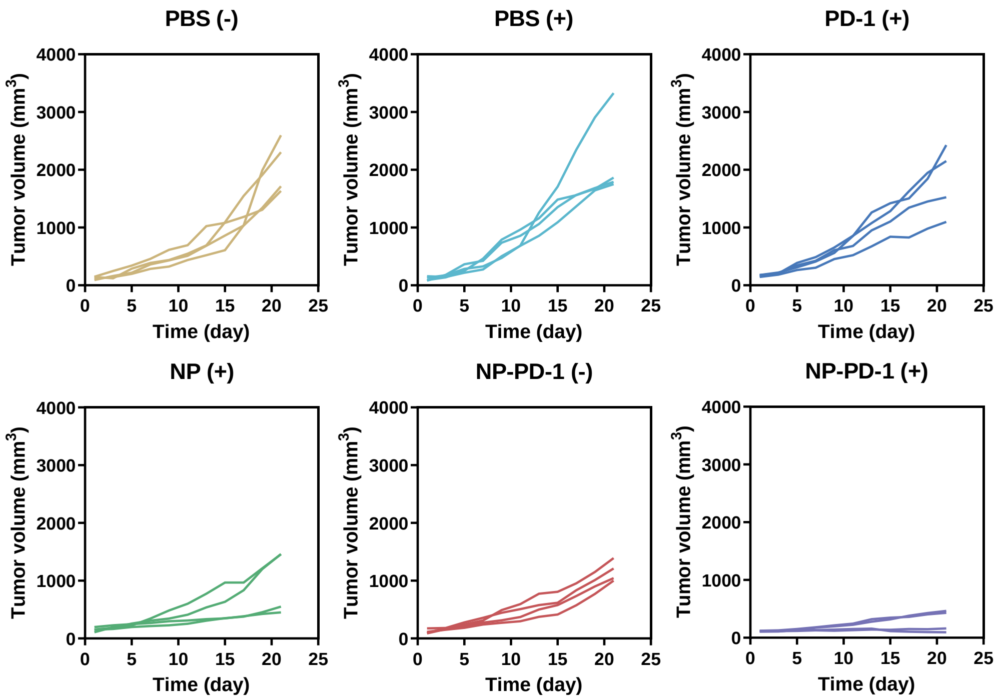

Figure S7. Primary tumor growth curves for orthotopic combined bilateral tumor model, n=4.

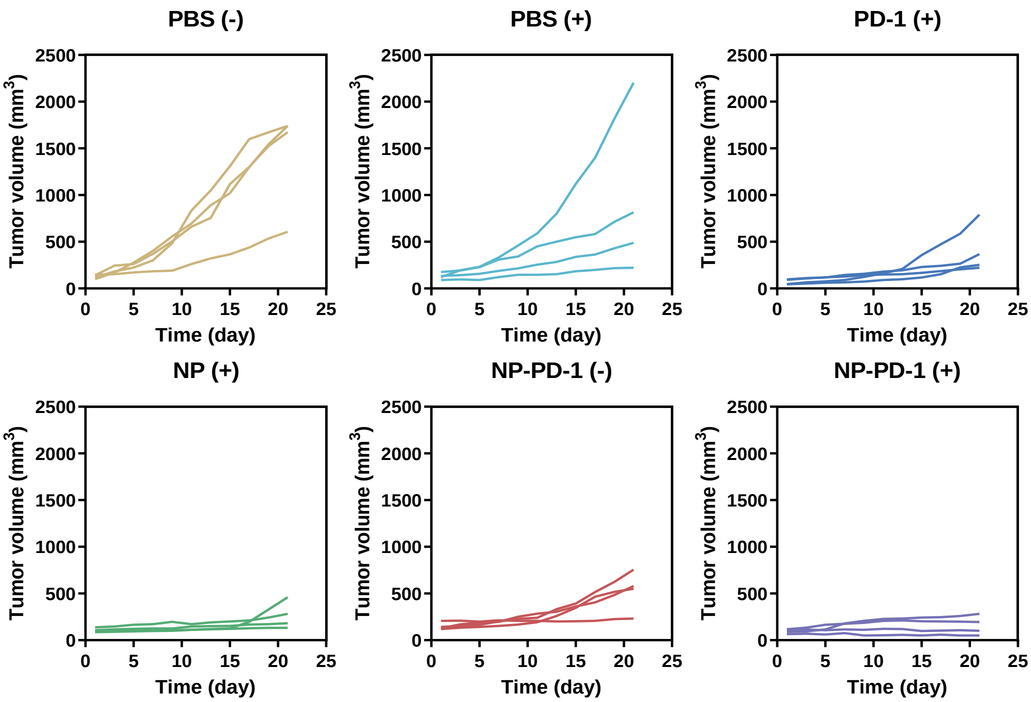

Figure S8. Distant tumor growth curves for orthotopic combined bilateral tumor model, n=4.

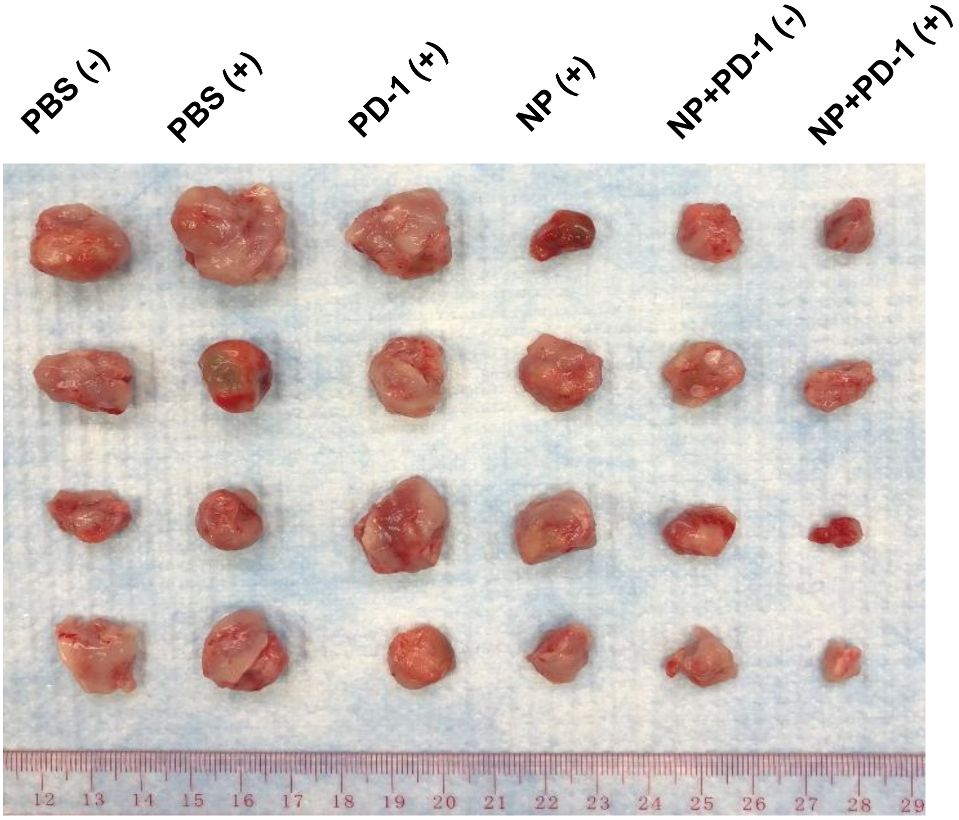

Figure S9. Photo of the primary tumors collected on day 21, n=4.

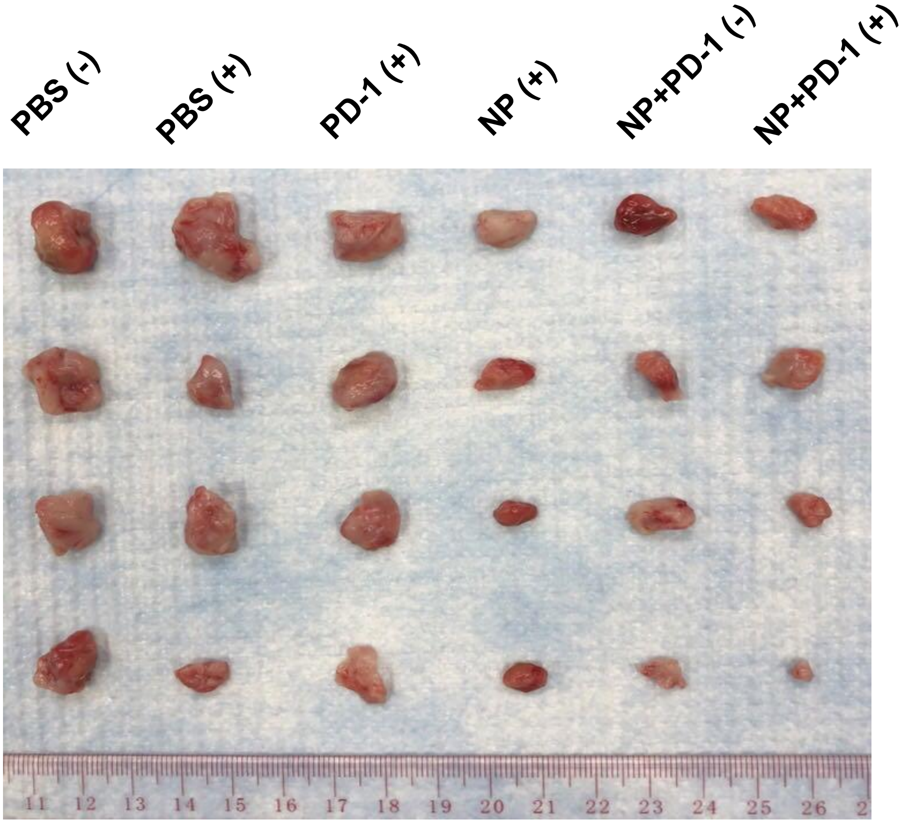

Figure S10. Photo of the distant tumors collected on day 21, n=4.

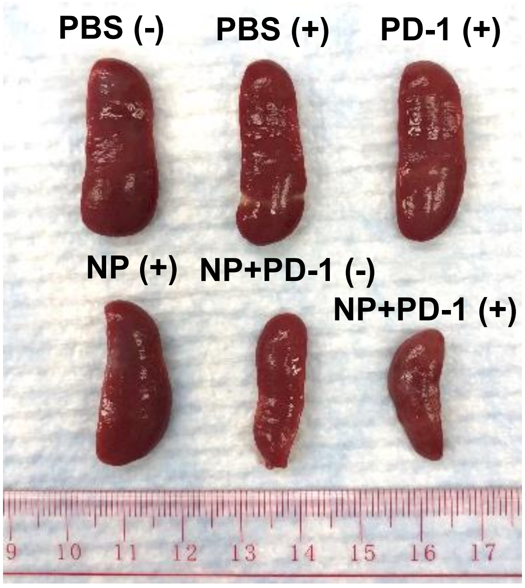

Figure S11. Photo of the spleen collected on day 21.

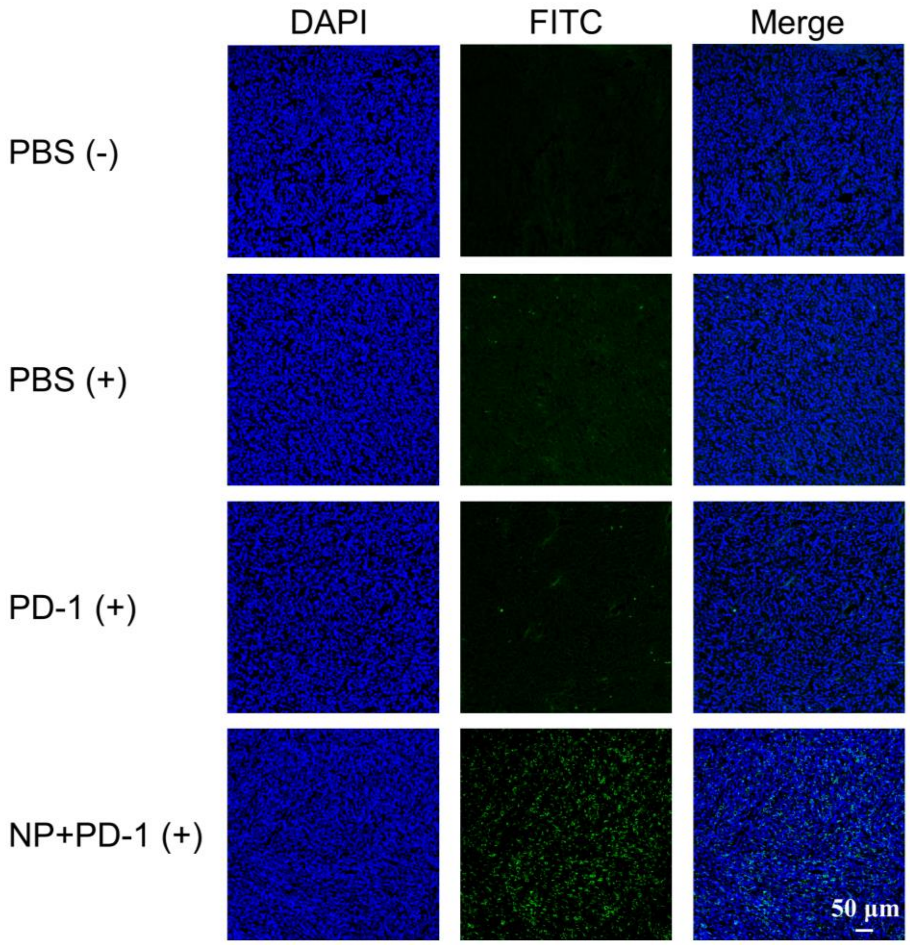

Figure S12. Representative TUNEL images of distant tumor slices after staining. Scale bar: 50 μm.

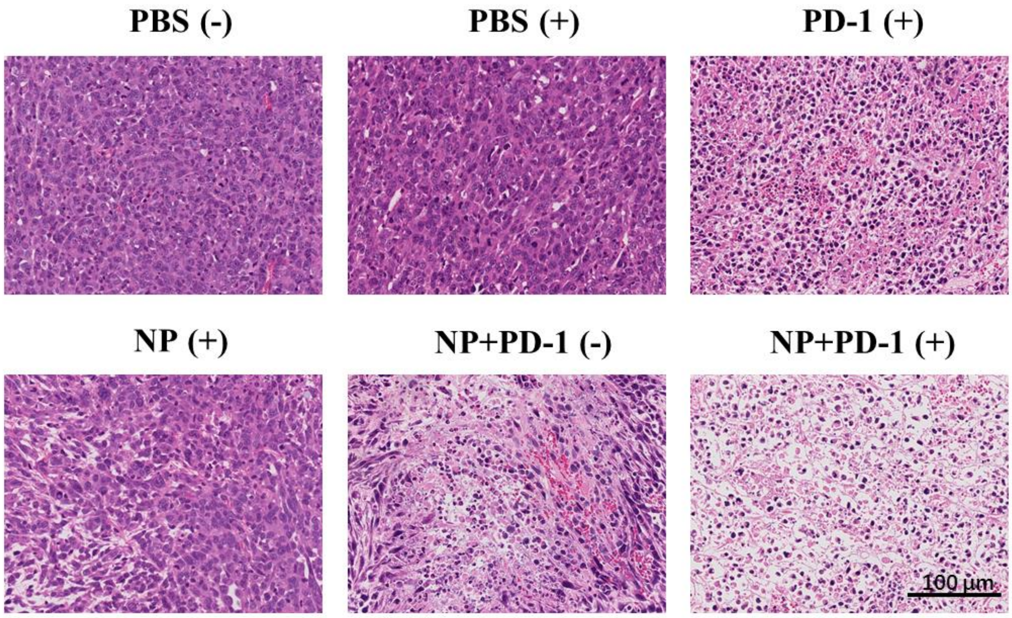

Figure S13. Hematoxylin and eosin (H&E)-stained slice images of distant tumor. Scale bar: 100 μm.

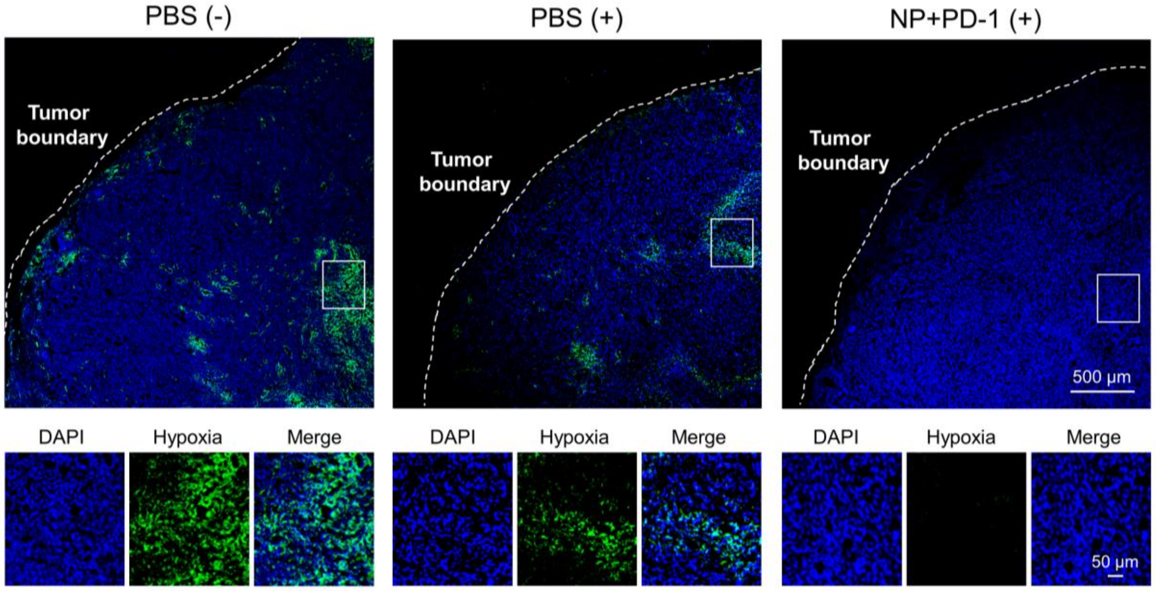

Figure S14. Representative immunofluorescence images of primary tumor slices after hypoxia staining. The hypoxia areas and nucleus were stained by HIF-α (green) and DAPI (blue), respectively.

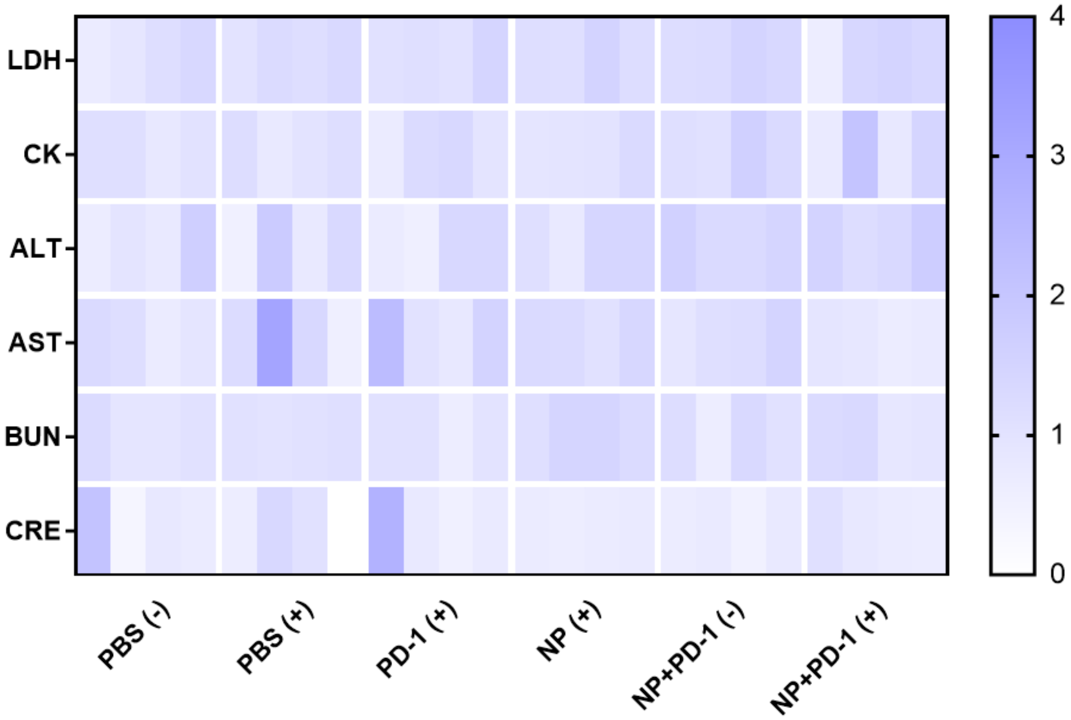

Figure S15. Biochemical parameters including lactate dehydrogenase (LDH), creatine kinase (CK), alanine aminotransferase (ALT), aspartate transaminase (AST), blood urea nitrogen (BUN) and creatinine (CRE), were determined. The color variance represents a ratio of the mice's biochemical data versus, n=4.

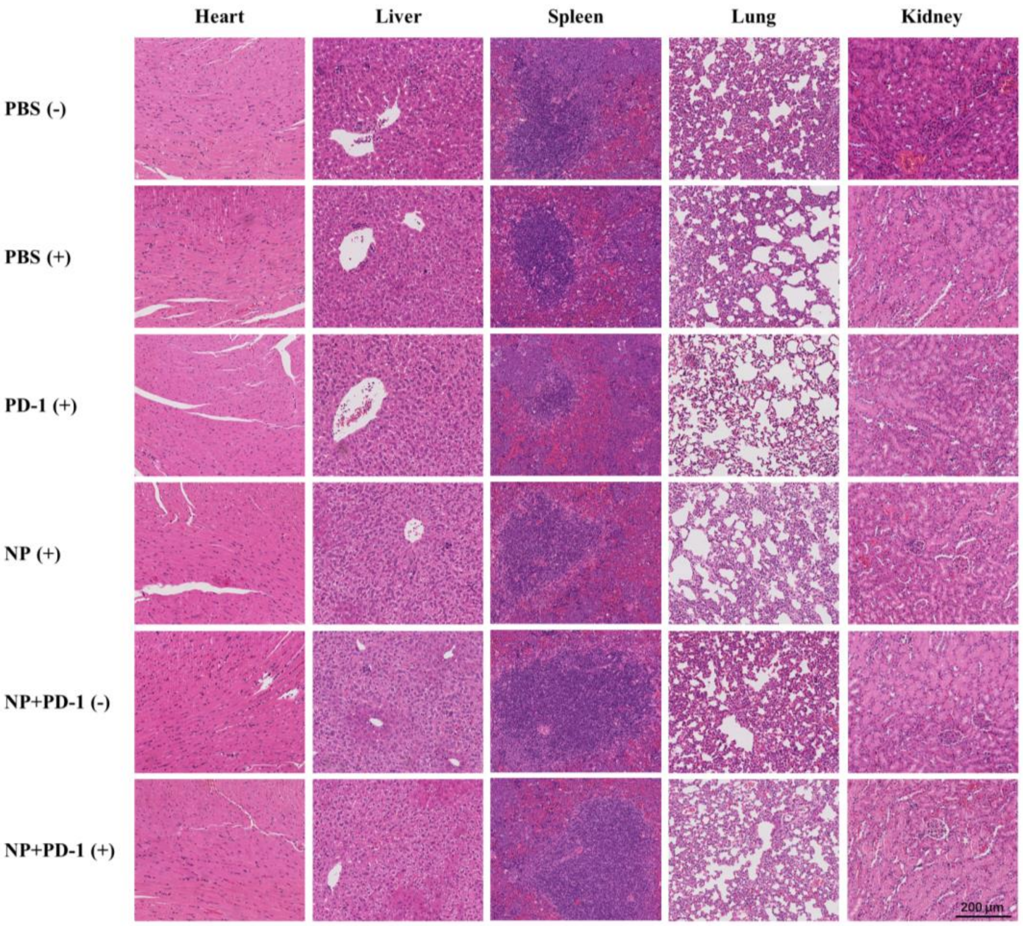

Figure S16. Hematoxylin and eosin (H&E)-stained slice images of heart, liver, spleen, lung and kidney, scale bar: 200 μm.

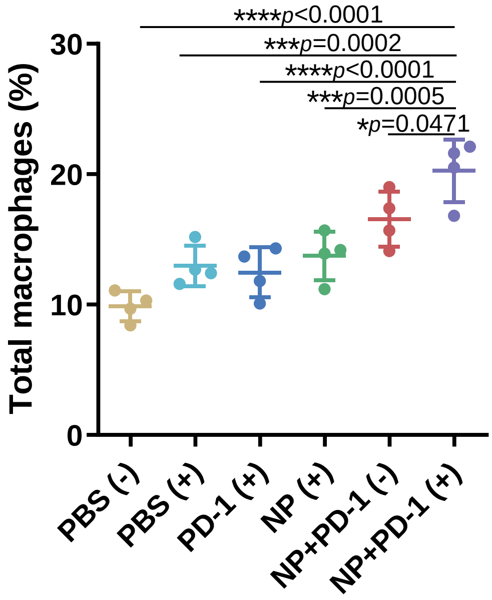

Figure S17. Proportions of total macrophages in primary tumors. Data are presented as mean values ± SD (n=4).

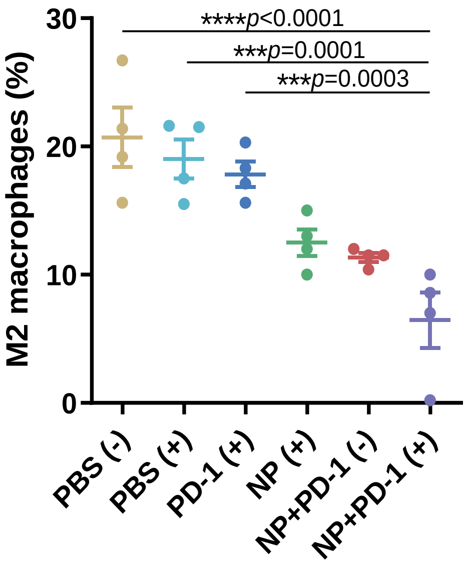

Figure S18. Proportions of M2 macrophages in primary tumors. Data are presented as mean values ± SD (n=4).

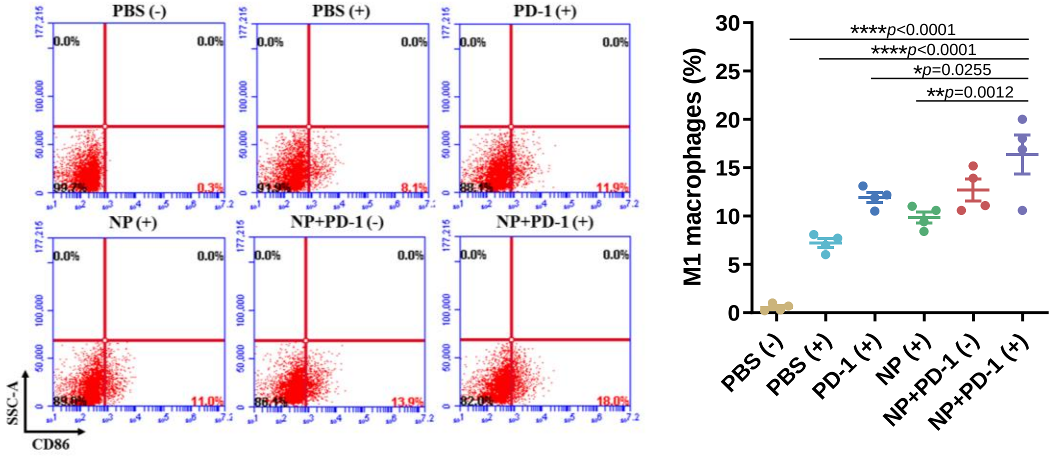

Figure S19. Representative flow cytometry plots and corresponding proportions of M1 macrophages in primary tumors. Data are presented as mean values ± SD (n=4).

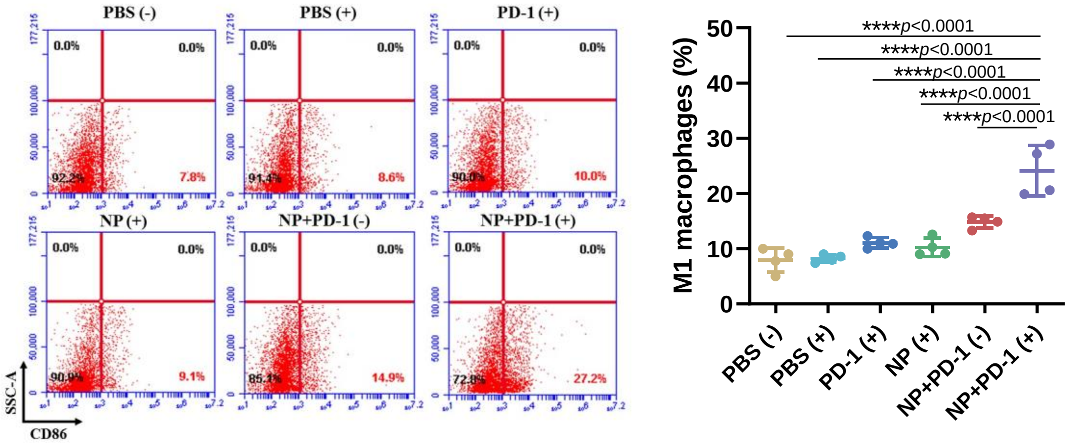

Figure S20. Representative flow cytometry plots and corresponding proportions of M1 macrophages in distant tumors. Data are presented as mean values ± SD (n=4).

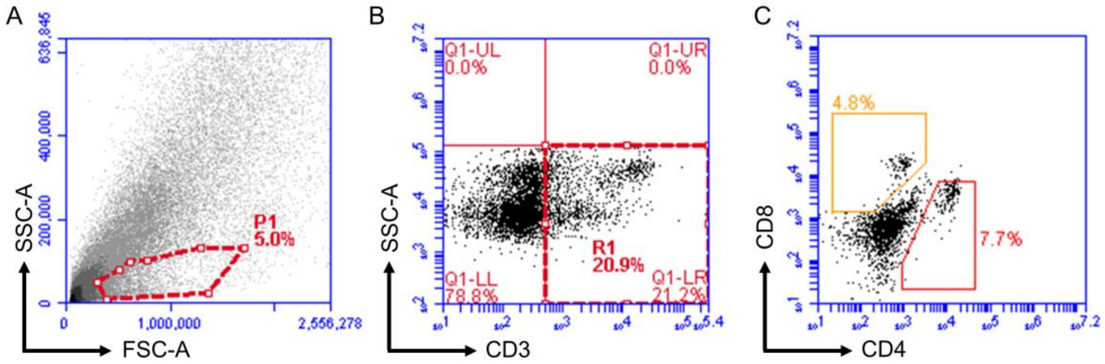

Figure S21. Gating strategies for flow cytometric analysis of T cells. (A) Gating total immune cells for P1 in solid tumors. (B) Gating $CD3^+$ T cells for R1 in total immune cells. (C) The $CD4^+$ and $CD8^+$ T cells in R1 gate are displayed in yellow and red boxes and the percentage of $CD4^+$ helper T cells ($CD4^+$ and $CD3^+$) and $CD8^+$ cytotoxic T cells ($CD8^+$ and $CD3^+$) is analyzed.

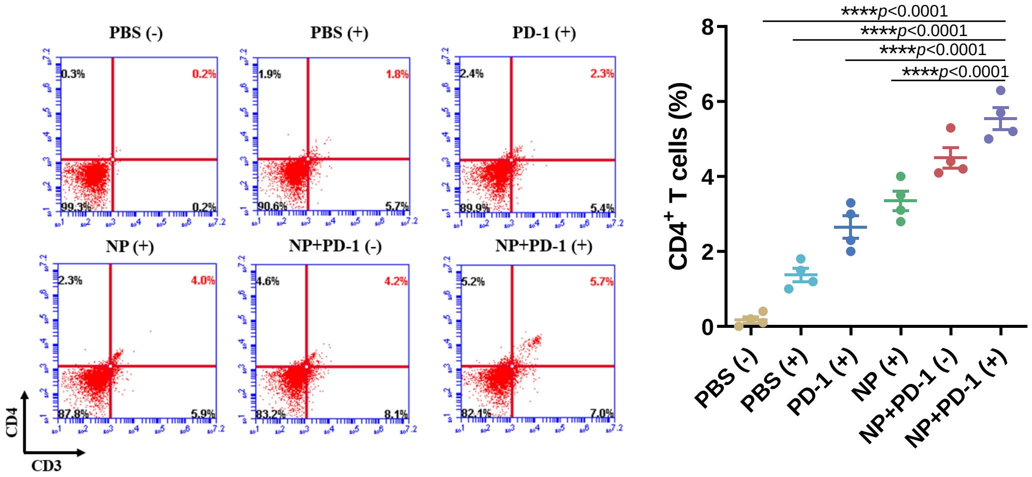

Figure S22. Representative flow cytometry plots and corresponding proportions of CD4$^+$ T cells in primary tumors. Data are presented as mean values ± SD (n=4).

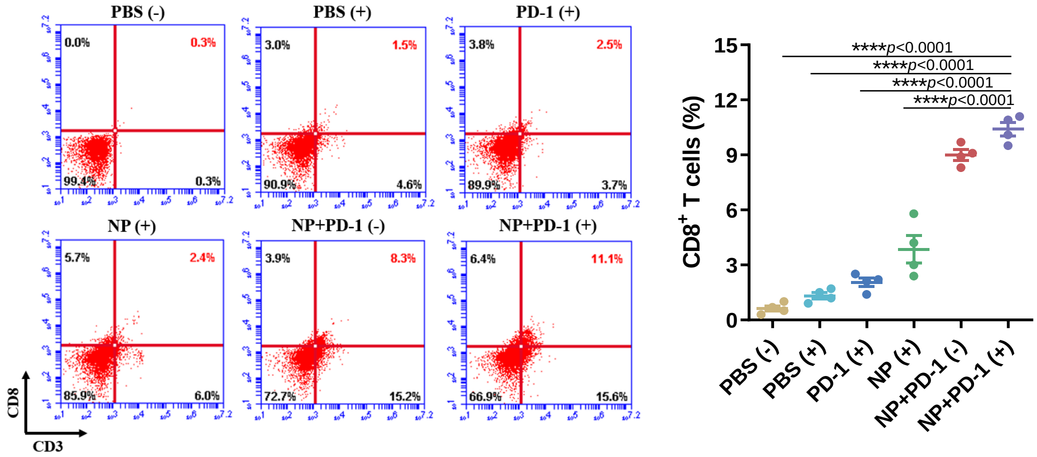

Figure S23. Representative flow cytometry plots and corresponding proportions of $CD8^+$ T cells in primary tumors. Data are presented as mean values ± SD (n=4).

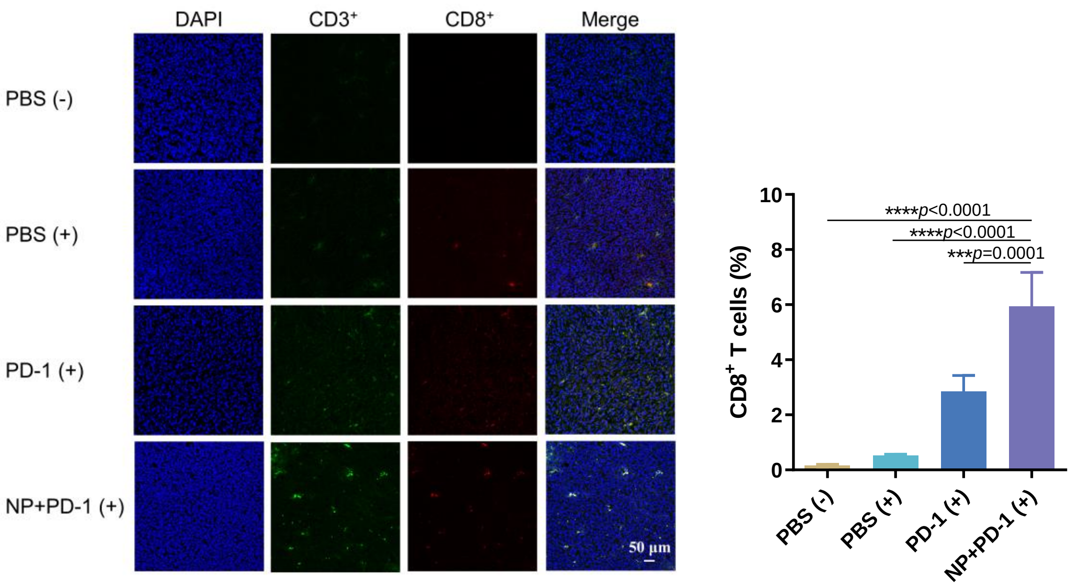

Figure S24. Representative immunofluorescence images and corresponding proportions of distant tumor slices after staining. The $CD8^+$ cells were stained by DAPI (blue), $CD3^+$(FITC) and $CD8^+$(Cy 5.5), respectively. Scale bar: 50 μm. Data are presented as mean values ± SD (n=4).

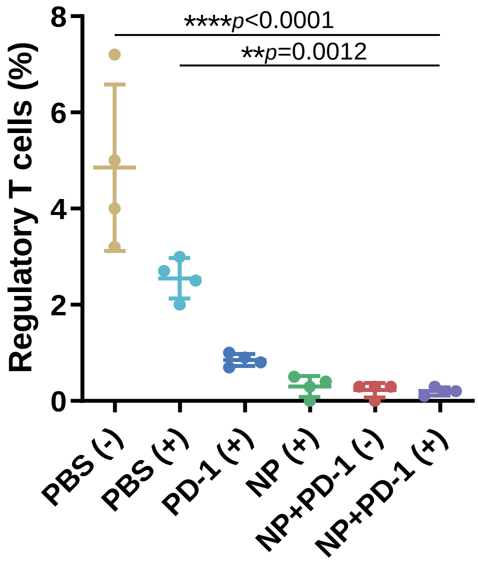

Figure S25. Proportions of regulatory T cells in primary tumors. Data are presented as mean values ± SD (n=4).

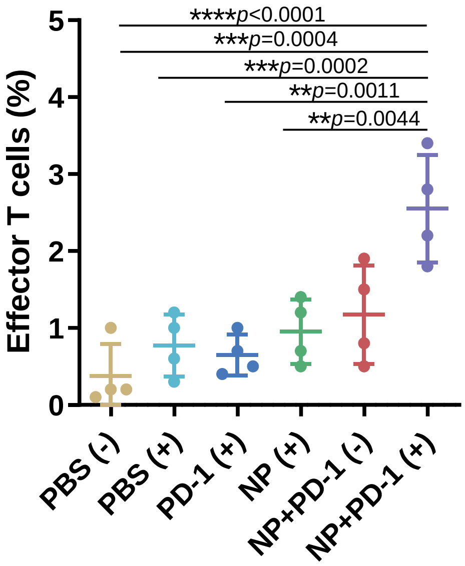

Figure S26. Proportions of effector T cells in primary tumors. Data are presented as mean values ± SD (n=4).

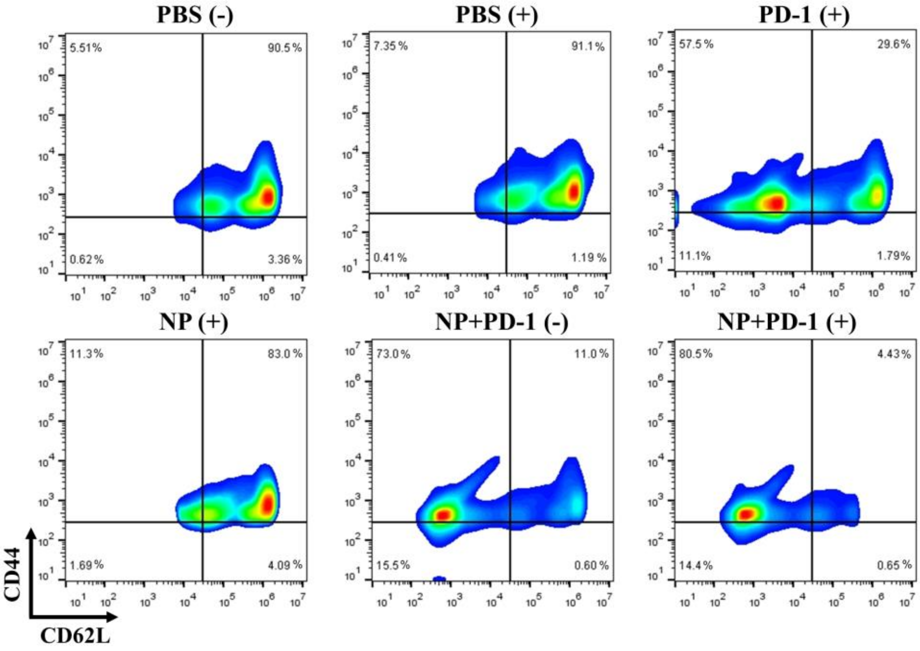

Figure S27. Representative flow cytometry plots of memory T cells in spleen.

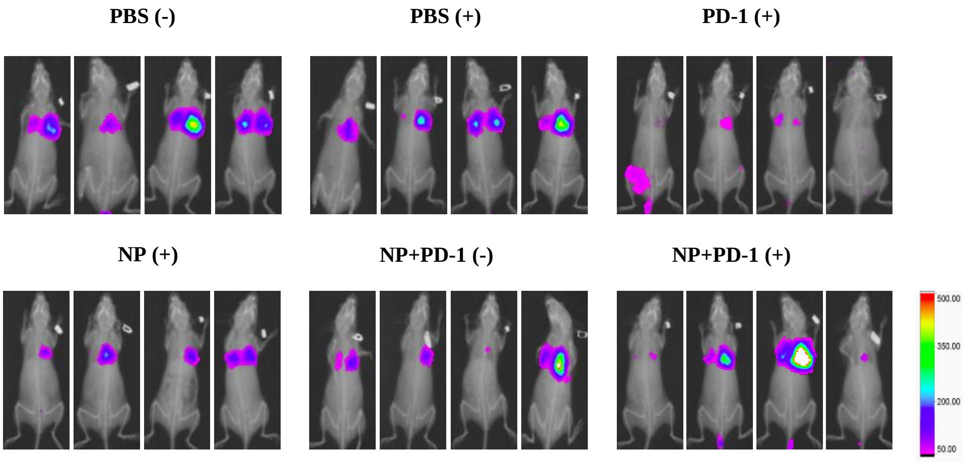

Figure S28. Bioluminescence images of lung metastasis in 4T1-luc cell-bearing mice on day 0, n=4.

Table S1. Pharmacokinetic parameters of Hb@Hf-Ce6 NPs in mice.

Parameter	Units	Mean	SD
t1/2α	h	1.006	0.220
t1/2β	h	19.313	4.807
AUC(0-t)	mg L^{-1}*h	126.273	19.514
AUC(0-∞)	mg L^{-1}*h	142.821	21.815
K10	h^{-1}	0.290	0.103
K12	h^{-1}	0.443	0.364
K21	h^{-1}	0.138	0.040
